# Vimentin–NF-κB signaling contributing to IbeA-mediated adhesion, invasion, and biofilm formation during *Escherichia coli* K1 traversal of the blood–brain barrier

**DOI:** 10.3389/fimmu.2026.1793594

**Published:** 2026-04-16

**Authors:** Bingliang Zhou, Xiangshun Meng, Zhengying Yu, Bao Zhang, Wei Zhao, Lei Wang, Liang Peng, Jinhu Zou, Jingyu Chen, Xu Lin, Xuefeng Gao, Sheng-He Huang, Hong Cao

**Affiliations:** 1 Department of Microbiology, Guangdong Provincial Key Laboratory of Tropical Disease Research, School of Public Health, Southern Medical University, Guangzhou, China; 2 Biological Safety Laboratory of Level 3 (BSL-3) Laboratory (Guangdong), Guangdong Provincial Key Laboratory of Tropical Disease Research, School of Public Health, Southern Medical University, Guangzhou, China; 3 Microbiome Medicine Center, Department of Laboratory Medicine, Zhujiang Hospital, Southern Medical University, Guangzhou, Guangdong, China; 4 Department of Clinical Laboratory, The Fifth Affiliated Hospital of Guangzhou Medical University, Guangzhou, Guangdong, China; 5 Department of Pediatrics, University of Southern California, Los Angeles, CA, United States

**Keywords:** biofilm, blood-brain barrier, *Escherichia coli* K1, IbeA, NF-κB, vimentin

## Abstract

**Introduction:**

IbeA is a critical virulence factor that is present in the GimA island in *Escherichia coli* (*E. coli*) K1, the most common cause of Gram-negative bacterial meningitis in newborns. IbeA has multiple virulence functions, including adhesion, invasion, and biofilm formation in the pathogenesis of *E. coli* K1 meningitis. However, it is unclear how IbeA coordinates its role in bacterial adhesion, invasion, and biofilm formation during crossing the blood-brain barrier (BBB).

**Methods:**

An isogenic *ibeA* deletion mutant (ZD1) was generated from the neonatal meningitis–associated *E. coli* K1 strain E44. To define IbeA-mediated virulence, bacterial adhesion, invasion, and biofilm formation were quantified in human brain microvascular endothelial cells (HBMECs) using TEER, confocal imaging, and crystal violet assays. A neonatal rat meningitis model evaluated bacterial dissemination, BBB disruption, neuroinflammation, and neurological deficits. Mechanistic studies focused on Vimentin (VIM) dynamics using lipid-raft fractionation, cytoskeleton extraction, immunofluorescence, vimentin-knockout (Vim-KO) HBMEC and Co-immunoprecipitation to assess its redistribution, post-translational modifications, and interaction with NF-κB. The functional involvement of the IbeA–VIM–NF-κB signaling pathway was further examined by modulating VIM activity with glatiramer acetate (GA) in both *in vitro* and *in vivo* experiments.

**Results:**

Deletion of *ibeA* and VIM significantly impaired bacterial adhesion, invasion, and biofilm formation both on HBMECs and on abiotic surfaces. In neonatal rats, infection with ZD1 resulted in higher survival rates, milder neurological symptoms, and a marked reduction of biofilm structures within the cerebral microvasculature compared with infection by the wild-type strain. IbeA-triggered post-translational modifications and cytoplasmic mobilization of VIM drive its nuclear translocation, which in turn activates NF-κB signaling. Pharmacological inhibition of VIM with GA disrupted IbeA-mediated virulence, lowering bacterial loads in the blood and alleviating neurological injury.

**Conclusions:**

IbeA is a key virulence factor that coordinates *E. coli* K1 adhesion, invasion, and biofilm formation by activating the VIM–NF-κB signaling pathway. Our study establishes for the first time a direct mechanistic link between IbeA’s multiple virulence functions through the above signaling pathway leading to acute BBB disruption and meningitis and identify GA as a potential drug development candidate for this disease.

## 1 Introduction

Bacterial meningitis continues to be a major global health problem in neonates. Among the causative agents, *Escherichia coli* (*E. coli*) K1 is the most common Gram-negative pathogen responsible for newborn meningitis (NMEC). Despite improvements in neonatal intensive care, NMEC is associated with a mortality rate of 10–15%, and 30–50% of survivors develop long-term neurological sequelae (1, 2). The situation is further complicated by the increasing prevalence of multidrug-resistant clones, such as *E. coli* O25-B2-ST131, which is recognized as a critically important superbug (3–5). Notably, these resistant strains often carry the *ibeA* gene, which was first identified by Blanco’s lab and Johnson’s group as a virulence factor associated with *E. coli* O25-B2-ST131 (6, 7).

Our previous work located *ibeA* within the GimA genomic island at the 98-min region of the *E. coli* K1 chromosome. At this locus, *ibeA* forms part of the regulatory *ibeRAT* operon and plays a central role in facilitating bacterial adhesion to and invasion of the blood-brain barrier (BBB) (8, 9). The presence of the complete GimA locus containing *ibeA* is almost exclusively seen in *E. coli* strains belonging to phylogenetic group B2 and is significantly more frequent in strains that cause newborn meningitis (NMEC) and avian pathogenic *E. coli* (APEC) (10, 11). IbeA was first identified by Germon’s group as an important virulence factor in APEC strains (12). Subsequent evidence from wang et al. found that IbeA-mediated biofilm formation could play an important role in the pathogenesis of avian pathogenic *E. coli* (13). Successful invasion of the central nervous system (CNS) requires a multi-step process: bacteria must adhere to, penetrate, and translocate across human brain microvascular endothelial cells (HBMECs), and then often establish robust biofilms that enable persistent infection (14). Both biofilm formation and BBB disruption are critical contributors to persistent infection and neurological damage (15, 16). However, the specific role of *ibeA* in coordinating these interconnected pathogenic processes remains defined.

We previously identified vimentin (VIM) as a key host receptor for IbeA (17, 18). In a seminal study, VIM was shown to undergo phosphorylation at Ser82 via CaMKII in *E. coli* K1 (IbeA^+^) invasion of HBMECs, establishing VIM phosphorylation as a functional prerequisite for bacterial entry (17). Subsequent studies demonstrated that IbeA binding to VIM and PSF activates NF-κB signaling, thereby facilitating bacterial invasion and neutrophil transmigration across HBMECs (19–21). Interestingly, vimentin has been found to be a novel upstream NF-κB regulator that is required for meningitis *E. coli* K1-induced bacterial Invasion across the BBB (21). These findings have demonstrated that the VIM–NF-κB signaling pathway contributes to both adhesion and invasion. It is unclear, however, that this signaling pathway contributes to biofilm formation. VIM has also been identified as a substrate for post-translational citrullination in contexts of glial activation and neuroinflammation (22), but whether VIM citrullination contributes to BBB disruption or bacterial meningitis pathogenesis remains unknown. Thus, whether IbeA mediates BBB disruption and biofilm formation through VIM-dependent NF-κB activation (and potentially other VIM modifications) has not been elucidated.

In this study, we investigate how IbeA plays a coordinated role adhesion, invasion, and biofilm formation during *E. coli* K1 infection. Secondly, we examine whether GA could serve as a novel inhibitor of vimentin and a new *in vitro* and *in vivo* tool for investigation of the VIM–NF-κB signaling pathway. Lastly, we examine if a single bacterial factor IbeA contributes to multiple virulence events through the common VIM–NF-κB signaling pathway that influences various stages of *E. coli* infection and disease development.

## 2 Materials and methods

### 2.1 Bacterial strains, cells, and culture conditions

E44 is a rifampicin-resistant derivative of the *E. coli* K1 strain RS218 (serotype O18:K1:H7), which was isolated from the cerebrospinal fluid of a patient with neonatal meningitis (23). ZD1 is an isogenic *ibeA* in-frame deletion mutant derived from E44 (9). Both strains have been used in our studies.

Human brain microvascular endothelial cells (HBMECs) were originally obtained from the laboratory of Dr. K. S. Kim and were provided as low-passage cell stocks (24). The cells were verified prior to experimental use to exhibit typical morphology and barrier properties characteristic of brain microvascular endothelial cells, consistent with previous reports (25). In addition, a VIM knockout (VIM KO) HBMEC cell line was recently constructed recently (26). Loss of VIM expression in this cell line was confirmed by Western blot prior to functional assays.

Bacteria were cultured in Luria–Bertani (LB) broth (Solarbio, China) supplemented with 50 µg/mL rifampicin. HBMECs were maintained in Dulbecco’s Modified Eagle Medium (DMEM, Gibco, USA) containing 10% fetal bovine serum (Procell, China) and 1% penicillin–streptomycin (Gibco, USA).

### 2.2 Bacterial adhesion and invasion assays

The adhesion and invasion assays were performed according to previously established methods (17, 27) with minor modifications. In brief, HBMECs were seeded in 24-well plates and grown to confluence. Stationary-phase *E. coli* was washed with sterile PBS and resuspended in antibiotic-free DMEM. Cell monolayers were infected at multiplicities of infection (MOI) of 1, 5, or 10 and incubated at 37 °C with 5% CO_2_ for 2 h. In parallel, uninfected HBMECs cultured in antibiotic-free DMEM under identical conditions were used as the control group. After incubation, cells were washed three times with pre-warmed PBS to remove non-adherent bacteria. For adhesion assays, adherent bacteria were detached using a cell scraper, serially diluted, and plated on LB agar for colony-forming unit (CFU) counting. For invasion assays, monolayers were treated with DMEM containing 100 µg/mL gentamicin for 1 h to eliminate extracellular bacteria, then lysed with 0.1% Triton X-100. Lysates were plated on LB agar to quantify intracellular CFUs. Adhesion and invasion efficiencies were calculated as the percentage of the initial inoculum that remained adherent or intracellular.

### 2.3 Biofilm formation assay on HBMECs

Biofilm formation on HBMECs was assessed as previously described with slight modifications (13). Briefly, HBMECs were seeded in 24-well plates and cultured to full confluence. Uninfected HBMECs cultured in antibiotic-free DMEM were included in parallel as the control group. Bacterial suspensions were adjusted to the desired concentration in antibiotic-free DMEM, and 500 μL was added per well at MOIs of 1, 5, and 10. The plates were incubated statically at 37 °C in 5% CO_2_ for 3, 6, or 9 h. At each time point, the wells were gently washed twice with PBS to remove planktonic bacteria. Adherent biofilms were fixed with 4% paraformaldehyde for 30 min and stained with 2% crystal violet (CV) for 20 min. After washing with distilled water and air-drying, biofilm structures were visualized and imaged under an inverted microscope at 20× and 40× magnifications (Nikon ECLIPSE Ti2, Japan). For quantitative analysis, the bound CV was solubilized with 33% glacial acetic acid, and absorbance was measured at 570 nm using a microplate reader.

### 2.4 Transepithelial electrical resistance measurement

HBMECs were seeded onto Transwell inserts and cultured for 12 hours until a stable monolayer with a TEER value of 200–250 Ω·cm² was formed. TEER was measured using an EVOM2 volt-ohm meter (World Precision Instruments, USA). TEER values of uninfected monolayers maintained under identical conditions were measured in parallel as the control group. Cells were then infected with bacteria at a multiplicity of infection (MOI) of 5. Based on preliminary optimization experiments evaluating bacterial concentration and differences between Transwell inserts and standard culture conditions, a 2.5-hour infection period was selected as the optimal time point to reliably detect changes in endothelial barrier integrity. TEER values were subsequently measured to assess BBB integrity following bacterial infection.

### 2.5 Immunofluorescence

For the analysis of bacterial biofilms and host cell signaling, immunofluorescence staining was performed. Uninfected HBMECs cultured under identical conditions served as the control group. For experiments involving pharmacological inhibitors, cells in the control group received an equal volume of the corresponding vehicle to exclude solvent-related effects. Biofilms formed on glass coverslips were fixed with 4% paraformaldehyde. For general structural visualization, the biofilms were stained with 5 µM DIO (UElandy, China) for 20 minutes to label bacterial membranes and counterstained with 1 µg/mL DAPI (Servicebio, China) for 15 minutes to label nucleic acids.

For the immunofluorescence staining of infected HBMECs, cell monolayers were fixed with 4% paraformaldehyde, permeabilized with 0.1% Triton X-100, and blocked with 5% bovine serum albumin (BSA) for 1 h at room temperature. The samples were then incubated overnight at 4 °C with the following primary antibodies: rabbit anti-vimentin recombinant monoclonal antibody (Proteintech, China; Cat. No. 80232-1-RR; 1:5000 dilution), mouse anti-phosphorylated vimentin (Ser82) monoclonal antibody (MBL, Japan; Cat. No. D095-3; 1:2000 dilution), mouse anti-citrullinated vimentin monoclonal antibody (Cayman Chemical, USA; Cat. No. 12G11; 1:2000 dilution), and mouse anti-NF-κB p65 monoclonal antibody (ZEN BIO, China; Cat. No. 380925; 1:1000 dilution). After washing with PBS, the samples were incubated for 1 h at room temperature with the appropriate fluorescent secondary antibodies: FITC-conjugated goat anti-rabbit IgG (H+L) and FITC-conjugated goat anti-mouse IgG (H+L) (Proteintech, China; Cat. No. SA00003-1 and SA00003-2; 1:200 dilution). Fluorescent images of biofilm architecture and intracellular protein localization were acquired using a Nikon A1R confocal microscope with 60× or 100× oil-immersion objectives.

### 2.6 Neonatal Rat *E. coli* K1 meningitis model

All animal experiments were approved by the Animal Ethics Committee of Southern Medical University (ethics project code: D2022090]). Five-day-old Sprague-Dawley rat pups were randomly assigned to experimental groups. The pups were infected intraperitoneally with 1×10^6^ CFU/10 g (28). Control animals received an equal volume of sterile normal saline via intraperitoneal injection following the same procedure and injection volume as infected groups and were defined as the control group. Neurological deficits were evaluated 24 hours post-infection using a modified neurological severity score (mNSS, **Supplementary Table S1**) (29). Survival was monitored for 72 hours. To assess bacterial load, blood and CSF were collected via cardiac puncture and cisternal puncture, respectively, at 24 hours post-infection. The samples were serially diluted and plated on LB agar for CFU counting. BBB disruption was evaluated by intraperitoneal injection of 2% Evans Blue (4 mL/kg). After 1 hour, the brains were harvested, and dye extravasation was quantified by measuring the OD_630_ of formamide-extracted dye.

### 2.7 Lipid raft extraction

Lipid rafts were isolated from HBMECs using the UltraRIPA Lipid Raft Extraction Kit (Applysgen, China) following the manufacturer’s protocol. Uninfected HBMECs processed under identical conditions were used as the control group. In brief, cells were first lysed with a detergent-free lysis buffer and centrifuged. The supernatant was discarded, and this lysis step was repeated once to thoroughly remove soluble cellular components. The resulting pellet was then resuspended and thoroughly lysed in the provided lipid raft extraction lysis buffer by repeatedly passing the solution through a 26-gauge needle. Following centrifugation, the low-density detergent-resistant membrane fraction (lipid rafts) was collected from the supernatant. Extracted lipid raft samples were mixed with SDS-PAGE loading buffer for Western blot analysis.

### 2.8 Soluble and insoluble VIM extraction

Soluble and insoluble VIM fractions extraction were extracted according to the protocol described by Luo (30). Parallel samples from uninfected HBMECs were included and served as the control group. Cells were lysed in buffer containing 2% sodium deoxycholate (Aladdin, China) and passed through a 26-gauge needle to facilitate lysis. The lysates were centrifuged at 18,400 × g for 20 minutes at 4 °C. The supernatant containing the soluble protein fraction was collected, and the pellet was resuspended in SDS-PAGE loading buffer as the insoluble protein fraction. These fractions were subsequently used for Western blot analysis.

### 2.9 Co-immunoprecipitation

Nuclear proteins were first extracted from HBMECs using a commercial Nuclear Protein Extraction Kit (Applygen, China). Uninfected HBMECs served as the control group. The extracted nuclear proteins were then incubated overnight at 4 °C with an anti-NF- κB p65 antibody. Subsequently, the immunocomplexes were captured using a commercial Co-IP Kit (MedChemExpress, China) according to the manufacturer’s instructions. Rabbit IgG (isotype-matched to the primary antibody, Proteintech, China, Cat No. 98136-1-RR) was used as a negative immunoprecipitation control. The captured complexes were thoroughly washed, eluted, and then subjected to Western blot analysis to detect the presence of VIM using the specified anti-VIM antibody.

### 2.10 Western blotting

Following infection with bacterial strains for the indicated durations, HBMECs were harvested and lysed to extract total cellular proteins. Uninfected HBMECs cultured in antibiotic-free DMEM under identical conditions served as the control group. For inhibitor experiments, equal volumes of the corresponding vehicle were added to the CON samples. The protein samples were separated by SDS-PAGE and transferred onto PVDF membranes (Merck Millipore, USA). After blocking, the membranes were incubated overnight at 4 °C with the following specific primary antibodies: anti-VIM, anti-Phosphorylated VIM (Ser82), anti-Citrullinated VIM (144-146), anti-p65, anti-Phospho-NF-κB p65 (Ser536) (Abmart, China; Cat. No. TP56372S; 1:2000 dilution), and anti-GAPDH (Proteintech, China; Cat No. 60004-1-Ig; 1:5000 dilution). The membranes were then incubated with horseradish peroxidase (HRP)-conjugated secondary antibodies (Proteintech, China; Cat No. SA00001-1and SA00003-2; 1:5000 dilution), and protein signals were visualized using an enhanced chemiluminescence detection system.

### 2.11 Statistical analysis

All data are presented as the mean ± standard deviation (SD) from at least three independent experiments. Statistical analyses were performed using SPSS 22.0 software. Differences between two groups were analyzed using Student’s *t*-test, and comparisons among multiple groups were performed using one-way analysis of variance (ANOVA) followed by Tukey’s *post-hoc* test. A *p*-value < 0.05 was considered statistically significant. Graphs were generated using GraphPad Prism software (version 8.0).

## 3 Results

### 3.1 IbeA facilitates E44 adhesion, invasion, and biofilm formation on HBMECs

**Figure 1:**
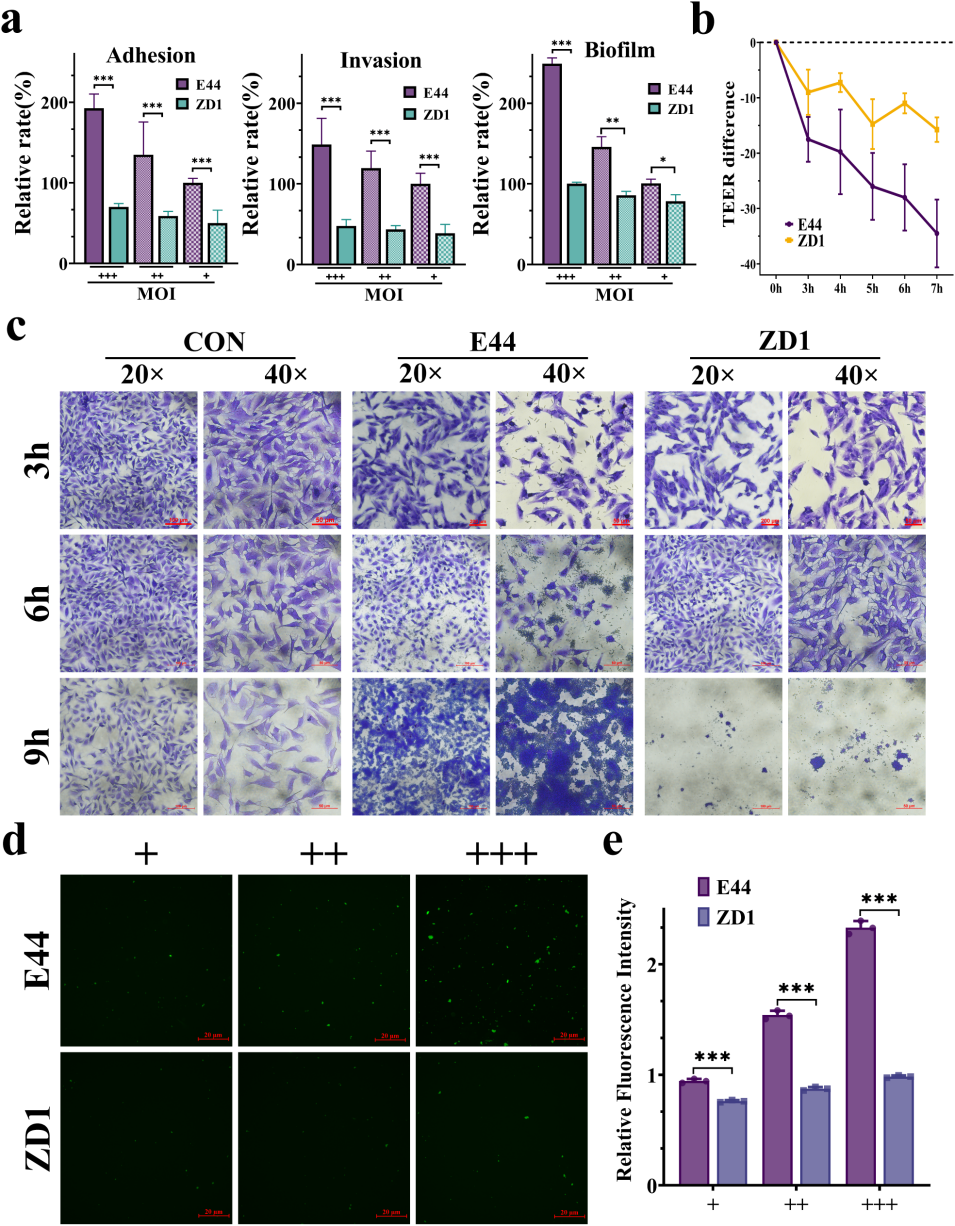
IbeA enhances *E. coli* K1 virulence on HBMECs. **(a)** Relative level of bacterial adhesion, invasion, and biofilm formation in E44 versus ZD1. **(b)** TEER measurements of HBMECs monolayers at different time points post-infection. **(c)** Representative micrographs of HBMECs stained with CV at 3, 6, and 9 h post-infection. Scale bars: 20× and 40×. **(d)** Fluorescence microscopy images of Hydrogel-based HBMECs cultures infected with GFP-tagged bacterial strains for 13 h. **(e)** Quantification of bacterial fluorescence intensity. Data are from three independent experiments (mean ± SD). *P < 0.05, **P < 0.01, ***P < 0.001.

To assess the contribution of IbeA to bacterial interactions with HBMECs, we compared the abilities of E44 and ZD1 to adhere to, invade, and form biofilms on HBMECs. Quantitative analysis revealed that deletion of *ibeA* significantly impaired all three virulence traits in a dose-dependent manner across MOI (**Figure 1a**; **Supplementary Figure S1a**). CV staining demonstrated that E44 exhibited extensive bacterial adhesion on HBMECs, surfaces as early as 3 hours post-infection, which progressively developed into microcolony aggregates and multilayered biofilm structures by 9 hours. In contrast, ZD1 infection resulted in sparse bacterial attachment at 3 hours, only modest increases by 6 hours, and limited clustering by 9 hours (**Figures 1a, c**). These observations indicate that IbeA plays a critical role in the initial adhesion and subsequent biofilm maturation during infection by *E. coli* K1.

Evaluation of the BBB integrity by TEER measurements showed that E44 infection induced a pronounced and sustained decline in TEER values over a 7-hour period, whereas ZD1 caused only a modest reduction (**Figure 1b**). Consistent with these results, E44 infection led to substantially greater HBMECs pathogenicity than the mutant strain (**Supplementary Figure S1b**). Hydrogel-based cell culture models and fluorescence microscopy further confirmed extensive E44 adhesion and clustering, whereas ZD1 exhibited weak signals (**Figure 1d**; **Supplementary Figure S1d**). Quantitative fluorescence analysis confirmed significantly stronger bacterial fluorescence intensity in E44-infected cultures than in ZD1 (**Figure 1e**). Together, these findings indicate that IbeA markedly enhances the ability of *E. coli* K1 to adhere to, invade, and form biofilms on HBMEC, thereby contributing to the compromise of the BBB integrity during infection.

### 3.2 IbeA increases meningitis pathogenicity by coordinating the three virulence effects of E44

**Figure 2:**
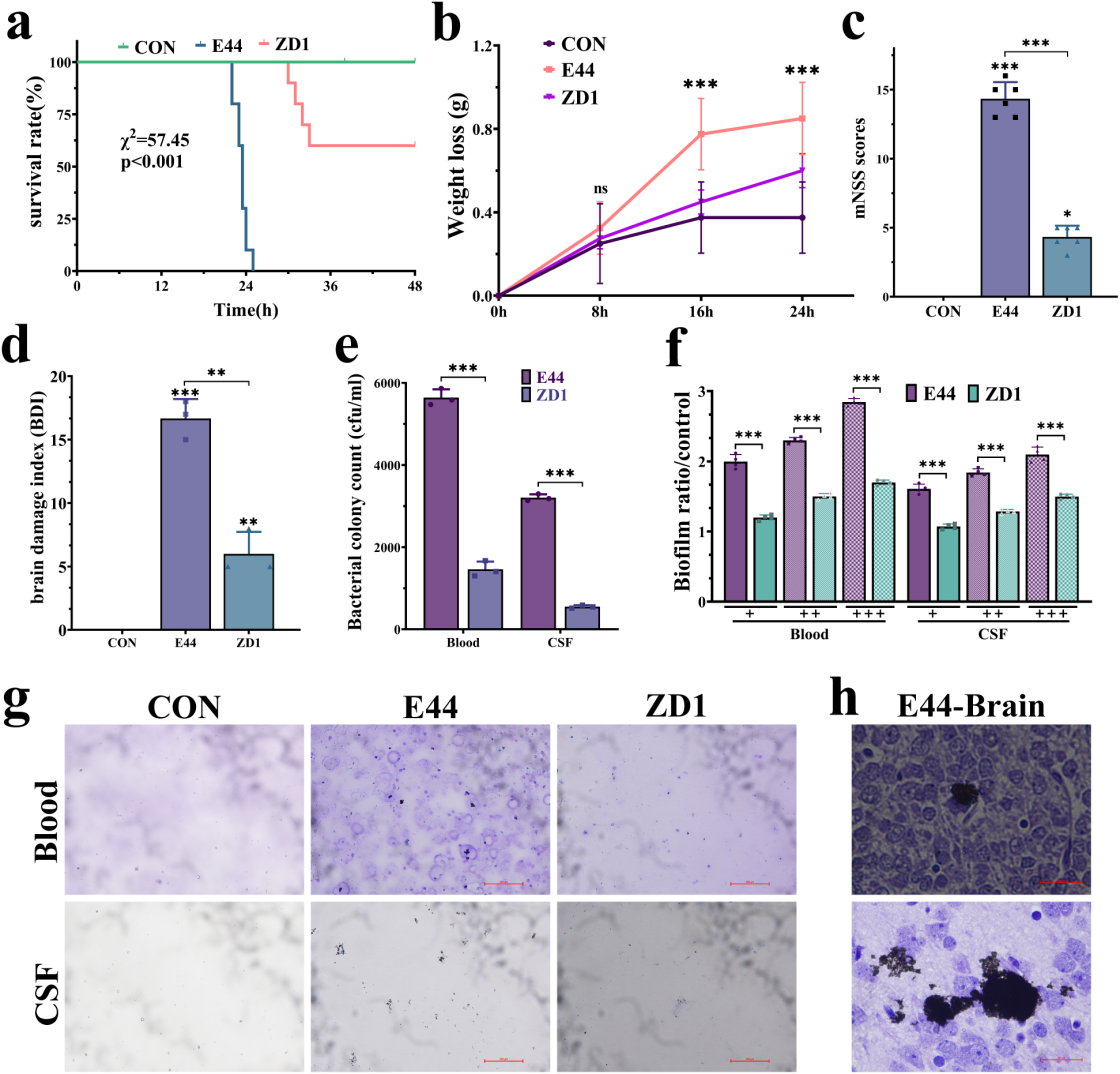
IbeA promotes virulence in neonatal *E. coli* K1 meningitis *in vivo*. **(a)** Kaplan–Meier survival curves. **(b)** Body weight changes of pups at indicated time points post-infection. **(c)** mNSS at 24 h post-infection. **(d)** BBB disruption index measured by Evans Blue extravasation. **(e)** Bacterial loads in blood and CSF. **(f)** Quantification of bacterial aggregation in blood and CSF. **(g)** Representative images of CV-stained blood and CSF samples. **(h)** Dense biofilm bacterial aggregates in brain tissue. Data are from six pups per group (mean ± SD). *P < 0.05, **P < 0.01, ***P < 0.001.

To extend our *in vitro* observations, we next evaluated the contribution of IbeA to the pathogenicity of *E. coli* K1 *in vivo* using a neonatal rat meningitis model. Kaplan–Meier survival analysis revealed markedly increased mortality in E44-infected pups, whereas *ibeA* deletion significantly improved survival (χ² = 57.45, *p <* 0.001) (**Figure 2a**). E44 infection caused pronounced weight loss, while ZD1 infection resulted in only mild reductions (**Figure 2b**). According to the mNSS scoring criteria, E44-infected neonates exhibited significantly elevated mNSS scores, reflecting severe motor dysfunction, delayed reflexes, and behavioral abnormalities, whereas ZD1-infected animals showed markedly lower scores, indicating attenuated neurotoxicity (**Figure 2c**).

Evans Blue permeability assays further demonstrated extensive BBB leakage in E44-infected brains, while ZD1 induced only modest increases in permeability (**Figure 2d**, **Supplementary Figure S2b**). Quantification of bacterial loads revealed significantly higher CFU counts in the blood and CSF of E44-infected animals than in those infected with ZD1 (**Figure 2e**). CV staining of blood and CSF samples showed extensive bacterial aggregates in the E44 group, in contrast to sparse clusters in ZD1 (**Figures 2f, g**). Histological examination of brain parenchyma confirmed dense biofilm bacterial aggregates and inflammatory infiltration in E44-infected animals, whereas ZD1-infected brains retained largely intact tissue architecture (**Figure 2h**; **Supplementary Figure S2**). Collectively, these results demonstrate that IbeA acts as a pivotal virulence determinant that promotes adhesion, invasion, biofilm formation, BBB disruption, and CNS colonization during neonatal *E. coli* K1 meningitis.

### 3.3 VIM serves as a key host regulator in IbeA-driven HBMEC

**Figure 3:**
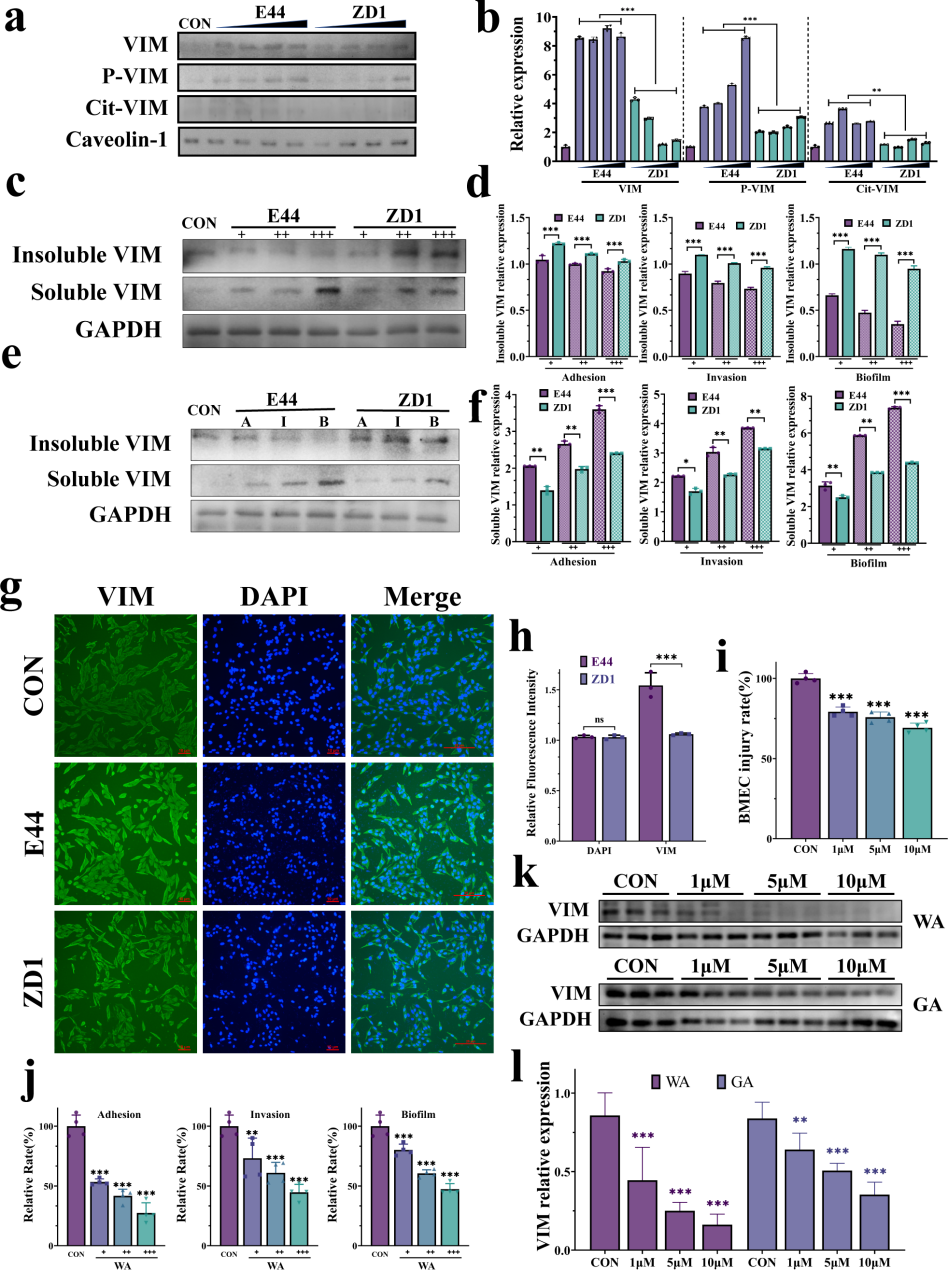
VIM mediates IbeA-induced pathogenicity. **(a, b)** VIM, phosphorylated VIM (Ser82) (P-VIM) and citrullinated VIM (144–146) (Cit-VIM) expression in lipid raft (Caveolin-1) fractions from HBMECs infected with E44 or ZD1. **(c, d)** Western blot analysis of VIM (54 kDa) and GAPDH (36 kDa) in soluble and insoluble protein fractions during adhesion, invasion, and biofilm formation. **(e, f)** Quantification of Western blot signals. **(g)** Immunofluorescence staining of HBMECs showing VIM (green) and nuclei (blue). **(h)** Quantitative analysis of VIM fluorescence intensity. **(i, j)** HBMECs infection after pretreatment with 1, 5, and 10 μM WA: cell pathogenicity **(i)** and relative adhesion, invasion, and biofilm formation **(j)**. **(k)** Western blot analysis of VIM expression in HBMECs treated with WA or GA. **(l)** Quantification of VIM protein levels from **(k)**. Data are from three independent experiments (mean ± SD). **P < 0.01, ***P < 0.001.

To identify host factors involved in IbeA-mediated multiple virulence functions, we examined the distribution and modification of VIM in lipid raft-enriched fractions of HBMECs following bacterial challenge. Lipid-raft isolation revealed a marked enrichment of VIM in lipid raft fractions upon infection with E44, whereas ZD1 failed to induce a comparable redistribution (**Figures 3a, b**, **Supplementary Figure S3**). Notably, VIM recovered in the raft fractions of E44-infected cells exhibited enhanced phosphorylation at Ser82 and citrullination at residues 144–146, indicating activation-associated post-translational modifications within membrane-associated microdomains and/or associated cytoplasmic pools. (**Figures 3a, b**). Western blot analysis further revealed that E44 infection elevated soluble VIM levels while reducing insoluble VIM fractions, indicating enhanced VIM mobilization and cytoskeletal remodeling. These alterations were not observed in ZD1-infected cells (**Figures 3c–f**). Consistently, immunofluorescence staining showed substantially stronger VIM signals in the cytoplasm and at the plasma membrane of E44-infected HBMECs compared with uninfected controls or ZD1-infected cells (**Figures 3g, h**).

To further validate the requirement of VIM in this process, VIM-KO HBMECs were generated. Western blot analysis confirmed the absence of VIM expression in VIM-KO cells, and neither E44 nor ZD1 infection was able to induce VIM upregulation in these cells (**Supplementary Figures S4a, b**). Functionally, VIM deficiency significantly reduced E44-mediated bacterial adhesion, invasion, and biofilm formation in HBMECs, whereas no significant differences were observed for the ZD1 strain (**Supplementary Figures S4c–e**). These findings further support that VIM is specifically required for IbeA-driven pathogenic processes.

Functionally, pharmacological inhibition of VIM with withaferin A (WA) significantly attenuated E44-induced HBMEC pathogenicity in a dose-dependent manner and markedly impaired bacterial adhesion, invasion, and biofilm formation (**Figures 3i, j**). These data demonstrate that VIM activation is critical for IbeA-mediated pathogenicity’s *in vitro*. Since WA displays strong toxicity in animals’ models this VIM inhibitor could not be used *in vivo*. To explore a safer and cost-effective drug repurposing strategy, we evaluated if GA, which was previously reported to downregulate VIM expression (31), could be used *in vivo* for the pathogenesis studies of meningitis. In line with this, GA treatment significantly suppressed VIM expression in HBMECs (**Figures 3k, l**) and effectively mitigated E44-induced pathogenic processes. Given the strong cytotoxicity of WA *in vivo*, GA represents a promising candidate for subsequent *in vivo* research tool. Collectively, these results further confirm that VIM is a critical mediator of IbeA-driven pathogenicity. IbeA induces the enrichment, phosphorylation, and citrullination of VIM within lipid rafts, promotes its cytoplasmic translocation, and facilitates *E. coli* K1 adhesion, invasion, and biofilm formation at the BBB.

### 3.4 IbeA induces post-translational modifications of VIM in the soluble fraction

**Figure 4:**
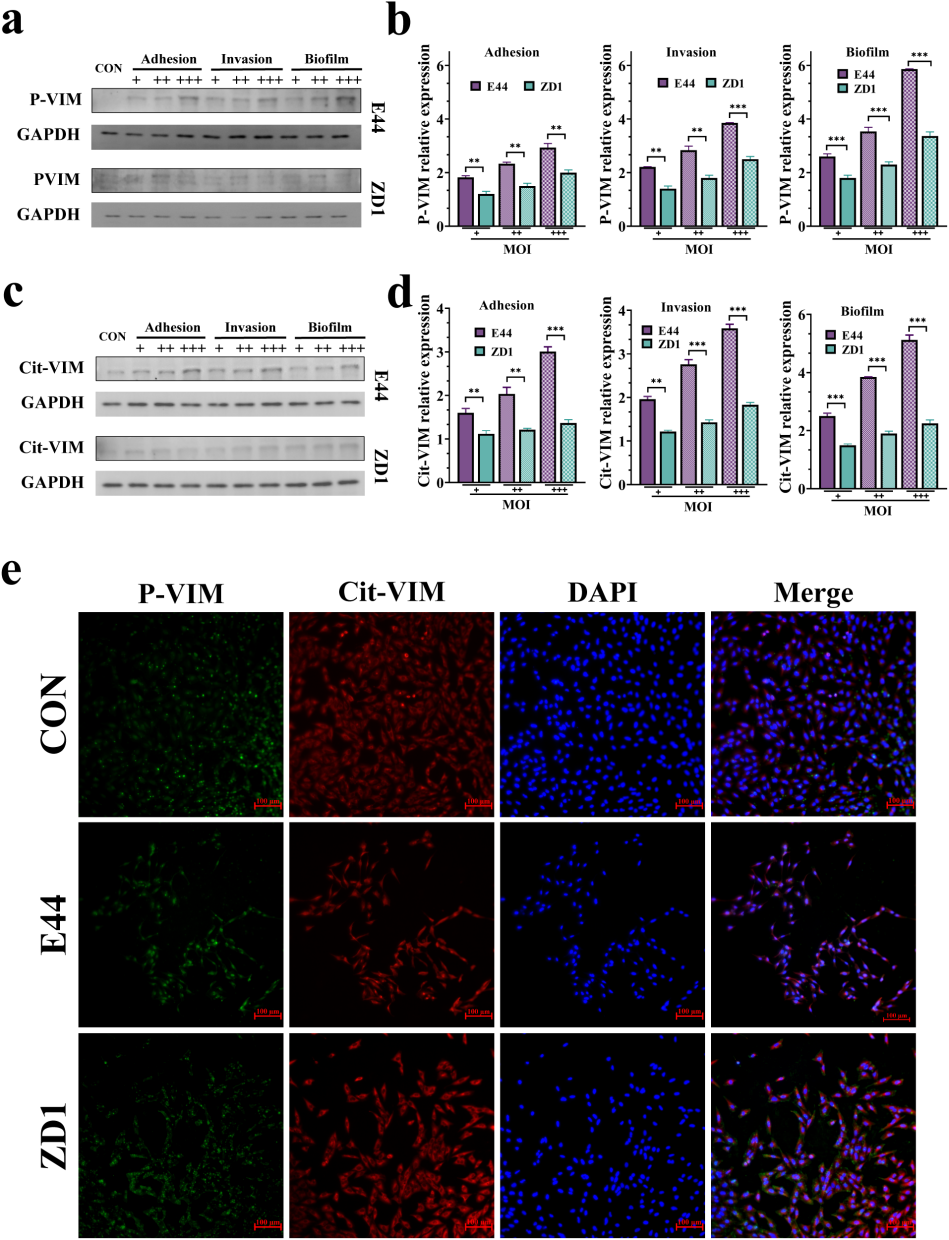
IbeA induces VIM post-translational modifications. **(a)** Western blot analysis of Phosphorylated VIM (Ser82) (54 kDa) and GAPDH (36 kDa) in soluble protein fractions during adhesion, invasion, and biofilm formation. **(b)** Quantification of Phosphorylated VIM (Ser82) expression. **(c)** Western blot analysis of Citrullinated VIM (144-146) (54 kDa) and GAPDH (36 kDa) in soluble protein fractions. **(d)** Quantification of Citrullinated VIM (144-146) expression. **(e)** Multiplex immunofluorescence staining of HBMECs showing Phosphorylated VIM (Ser82) (green), Citrullinated VIM (144-146) (red), and nuclei (blue). Data are from three independent experiments (mean ± SD). **P < 0.01, ***P < 0.001.

As lipid-raft analysis indicated increased that VIM abundance was increased with enhanced phosphorylation and citrullination after E44 infection, we next assessed whether these modifications were detectable in the soluble cytoplasmic fraction. Western blot analysis revealed that E44 infection produced a marked increase in both phosphorylated VIM (Ser82) and citrullinated VIM (144–146) in the soluble fraction of HBMECs through the adhesion, invasion, and biofilm formation stages (**Figures 4a–d**). Infection with ZD1 elicited changes in the same modified sites of VIM species, but these alterations were consistently smaller in magnitude compared with those induced by E44.

To evaluate the functional relevance of these post-translational modifications, we tested if these modifications depended on their regulatory enzymes. Pharmacological blockade of peptidyl arginine deiminases (PADs) with Cl-amidine or inhibition of CaMKII with KN-93 significantly impaired E44-driven adhesion, invasion, and biofilm formation; the inhibitory effects in ZD1-infected cells were present but less pronounced (**Supplementary Figure S5**). These data indicate that both citrullination and phosphorylation of VIM contribute to IbeA-dependent pathogenicity. Immunofluorescence analysis corroborated the biochemical results, showing notably higher levels of phosphorylated and citrullinated VIM within the cytoplasm of E44-infected HBMECs, whereas ZD1-infected and uninfected cells exhibited weaker baseline signals (**Figure 4e**). These findings indicate that coordinated phosphorylation and citrullination of VIM are key molecular events driving *E. coli* K1 adhesion, invasion, and biofilm formation.

### 3.5 IbeA-activated VIM functions as an NF-κB activator to promote HBMEC injury and bacterial pathogenesis

**Figure 5:**
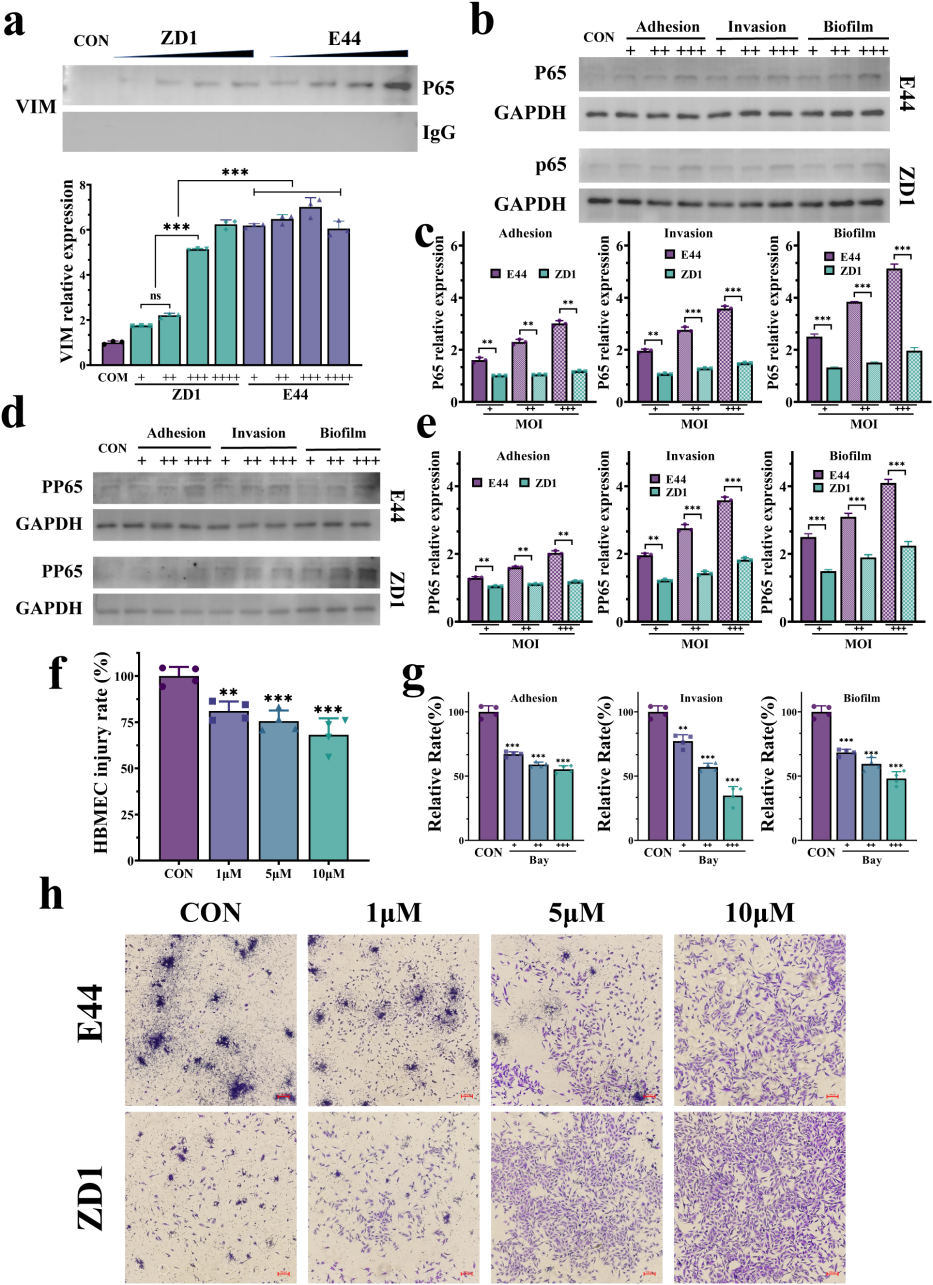
NF-κB acts downstream of IbeA-VIM signaling. **(a)** Co-IP analysis of VIM-p65 interaction in HBMECs infected with indicated strains. **(b)** Western blot analysis of p65 (65 kDa) and GAPDH (36 kDa) in soluble fractions during adhesion, invasion, and biofilm formation. **(c)** Quantification of p65 expression. **(d)** Western blot analysis of Phospho-NF-κB p65 (Ser536) (65 kDa) and GAPDH (36 kDa) in soluble fractions. **(e)** Quantification of Phospho-NF-κB p65 (Ser536) expression. **(f, g)** HBMECs infection after pretreatment with 1, 5, and 10 μM BAY: cell pathogenicity **(f)** and relative adhesion, invasion, and biofilm formation **(g)**. **(h)** Effects of BAY inhibition on HBMECs damage and biofilm formation. Data are from three independent experiments (mean ± SD). **P < 0.01, ***P < 0.001.

Since IbeA induces post-translational modifications and cytoplasmic mobilization of VIM, we investigated whether this modified VIM functions as an upstream activator of the master regulator NF-κB. Co-IP assays demonstrated a direct interaction between VIM and NF-κB, which was significantly enhanced in E44-infected HBMECs compared to ZD1-infected or uninfected controls (**Figure 5a**). In line with this finding, E44 infection markedly increased total and phosphorylated p65 levels during bacterial adhesion, invasion, and biofilm formation, whereas ZD1 infection resulted in only minimal NF-κB activation (**Figures 5b–e**).

To further determine whether VIM is required for NF-κB activation, we examined p65 expression in VIM-KO HBMECs following bacterial infection. Western blot analysis revealed that VIM deficiency markedly reduced p65 expression in E44-infected cells compared with wild-type HBMECs (**Supplementary Figures S6a, b**), indicating that VIM is necessary for efficient NF-κB activation during E44 infection.

**Figure 6:**
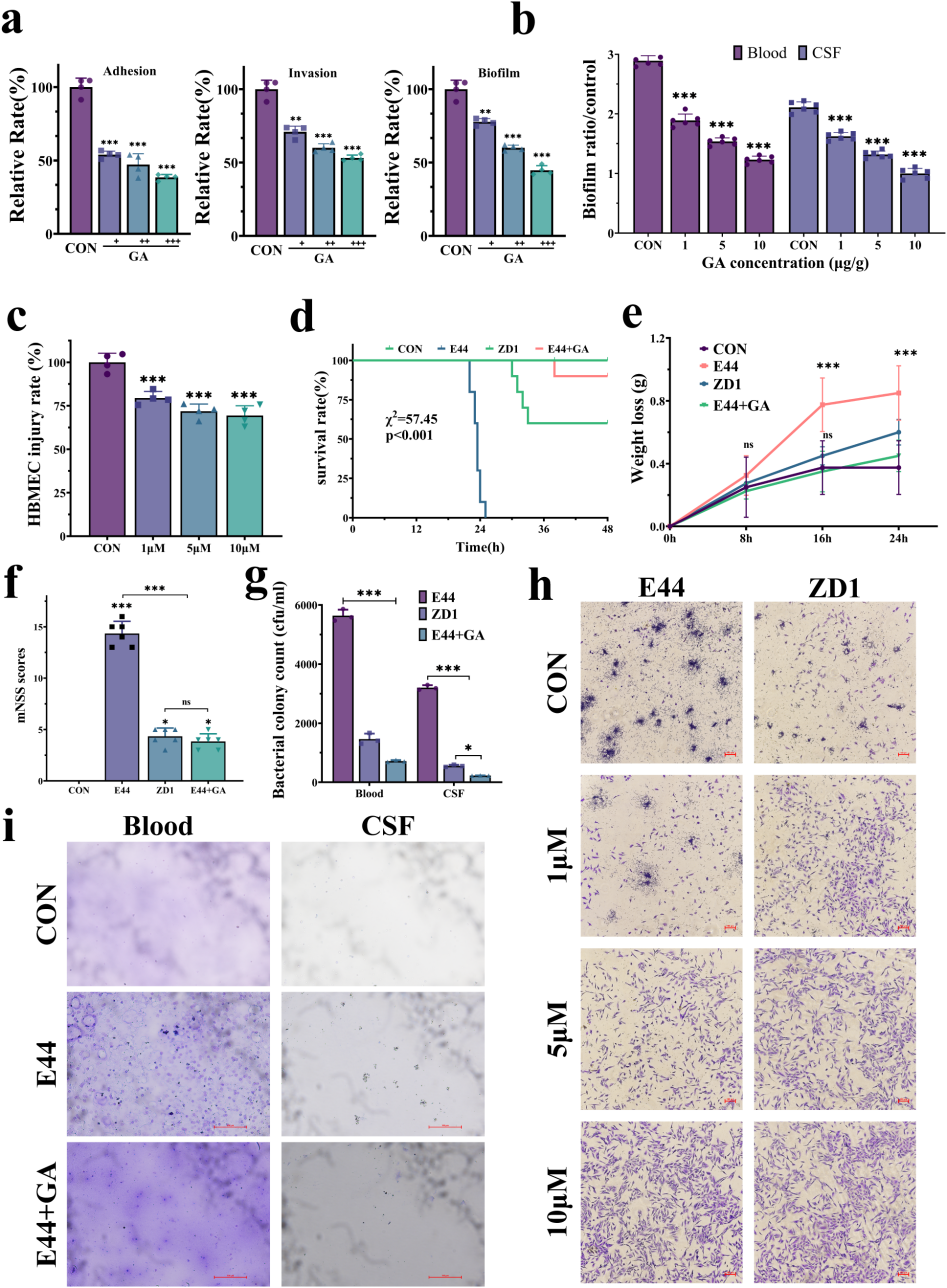
GA attenuates infection via VIM inhibition. **(a)** Relative adhesion, invasion, and biofilm formation in HBMECs pretreated with 1, 5, and 10 μM GA. **(b)** Quantification of bacterial aggregation in blood and CSF after GA treatment. **(c)** Cell pathogenicity analysis after GA pretreatment. **(d)** Survival curves of neonatal rats receiving GA treatment. **(e)** Body weight changes in GA-treated pups. **(f)** mNSS in GA-treated pups. **(g)** Bacterial loads in blood and CSF after GA treatment. **(h)** GA effects on HBMECs pathogenicity and biofilm formation. **(i)** CV staining of blood and CSF samples from GA-treated animals. Data are from three independent experiments *in vitro* and six pups per group *in vivo* (mean ± SD). ns, not significant; *P < 0.05; **P < 0.01; ***P < 0.001.

To further confirm if VIM served as the upstream activator, we used two pharmacological agents. Two VIM inhibitors WA and GA substantially reduced p65 phosphorylation and nuclear accumulation (**Supplementary Figures 6c, d**). Accordingly, both treatments significantly attenuated E44-induced HBMEC pathogenicity and suppressed bacterial adhesion, invasion, and biofilm formation (**Figures 3j**, **6a**). Although WA was more potent, GA exhibited superior cellular tolerance, highlighting its potential use as a research tool in animal models. As a complementary approach, a direct NF-κB inhibition with BAY 11-7082 also alleviated HBMEC injury and impaired bacterial pathogenicity in a dose-dependent manner (**Figures 5f–h**). However, the protective effects of BAY 11-7082 were less pronounced than those achieved by suppressing the upstream VIM activator. Collectively, these data define a pathogenic cascade in which IbeA-induced VIM activation drives NF-κB signaling to promote inflammatory responses and facilitate bacterial pathogenesis.

### 3.6 Host-directed therapy targeting VIM attenuates infection

Given the reported pleiotropic effects of WA that limit its suitability for *in vivo* applications, and to identify a more specific and tolerable VIM-targeting strategy, we next evaluated the use of GA as an integrated *in vitro* and *in vivo* research tool. Western blot analysis confirmed that GA treatment significantly reduced VIM expression in HBMECs without affecting cell viability, as verified by CCK-8 assay (**Figure 3k**, **Supplementary Figure S7a**). Functionally, GA treatment dose-dependently alleviated E44-induced HBMEC injury and markedly suppressed bacterial adhesion, invasion, and biofilm formation *in vitro* (**Figures 6a, c, h**), indicating that GA effectively counteracts IbeA-dependent pathogenicity through VIM suppression.

To further determine whether the protective effects of GA depend on VIM, VIM-KO HBMECs were pretreated with GA prior to E44 infection. In contrast to wild-type cells, GA treatment did not further reduce bacterial adhesion, invasion, or biofilm formation in VIM-deficient cells, and no significant differences were observed between GA-treated and untreated VIM-KO groups (**Supplementary Figure S7d**). These results indicate that the anti-pathogenic effects of GA are specifically mediated through VIM, supporting VIM as the primary functional target of GA in this context.

We further assessed the therapeutic efficacy of GA in a neonatal rat model of meningitis caused by *E. coli* K1. GA administration significantly improved survival (80% in the GA-treated group vs. 20% in the untreated controls, χ² = 57.45, *p <* 0.001; **Figure 6d**), mitigated infection-associated weight loss (**Figure 6e**), and significantly reduced mNSS scores compared with the untreated infected animals, indicating substantial improvement in neurological function (**Figures 6f, g**). Histopathological analysis revealed well-preserved brain parenchyma with minimal inflammatory infiltration and absence of biofilm bacterial aggregates. Consistently, CV staining of blood samples showed substantially reduced bacterial clustering in GA-treated animals (**Figures 6b, i**). Collectively, these findings demonstrate that GA is a promising host-directed therapeutic agent targeting VIM (**Supplementary Figures S7b, c**), which effectively mitigates IbeA-dependent bacterial pathogenicity both *in vitro* and *in vivo*, offering a potential intervention strategy against *E. coli* K1 meningitis.

## 4 Discussion

IbeA is a critical virulence factor that is encoded by the *ibeRAT* regulon on the genomic island GimA in *E. coli* K1, the most common cause of gram-negative NMEC. It has multiple virulence functions, including adhesion, invasion, and biofilm formation in the pathogenesis of *E. coli* K1 meningitis. However, it remains elusive how IbeA coordinates its role in mediating multiple virulence functions. In this study, we demonstrate that IbeA is an important virulence determinant that coordinates its multiple virulence functions in a manner dependent on VIM that is the upstream NF-κB activator.

Our *in vitro* and *in vivo* findings reaffirm the essential role of IbeA in traversal across the BBB while substantially extending its known functional repertoire. Here, we show that IbeA also governs the formation of robust biofilm structures on HBMEC surfaces, promoting bacterial persistence and immune evasion. In the neonatal rat model, the wild-type E44 strain formed conspicuous biofilm aggregates within brain parenchyma, whereas the *ibeA* deletion strain ZD1 failed to do so, highlighting that IbeA enables successful colonization and persistence on surfaces during the biofilm life cycle. Given the established roles of biofilms in antimicrobial tolerance and host immune modulation (32–34), these findings position IbeA as a unifying virulence factor that orchestrates multiple phases of CNS infection, extending classical models of IbeA function (35) and reflecting the multifaceted nature of virulence determinants in host–pathogen interactions (36–38).

Central to this coordinated virulence functions are VIM-mediated complex and multicomponent regulatory systems—specifically VIM acting as an upstream transcriptional regulator of NF-κB. Both chemical blocking and gene knockout of VIM are able significantly abolish IbeA-mediated virulence and VIM-mediated signaling. Previous studies from our group established VIM as the primary IbeA receptor on the HBMEC surface and demonstrated that IbeA–VIM engagement activates canonical NF-κB signaling to promote bacterial invasion across the BBB (20, 21, 39). In the present work, we expand this mechanistic paradigm by showing that IbeA stimulates not only VIM engagement but also its post-translational modification (phosphorylation and citrullination) and cytoplasmic mobilization. The multicomponent activation and signaling leads to form VIM–p65 complexes detectable within both cytoplasmic and nuclear compartments, thereby facilitating NF-κB activation, recruitment and amplifying downstream transcriptional responses. Such intracellular scaffolding functions indicate that VIM acts not simply as the primary receptor for IbeA but as an upstream signal transducer to activate NF-κB, which results in increased production of inflammatory effectors (21, 40, 41).

This refined mechanistic model further supports the pathogenic triad of neonatal meningitis—characterized by bacterial invasion, neutrophil transmigration across the BBB and host NF-κB activation—as originally proposed in studies of VIM-mediated signaling (17, 39). Our data demonstrate that the IbeA–VIM–NF-κB axis governs not only invasion but also later-stage events such as biofilm development. Through NF-κB–dependent transcriptional activation of adhesion molecules, inflammatory mediators, and cytoskeletal regulators, endothelial cells create a microenvironment that supports microcolony formation and matrix stability (42, 43). This reveals a previously unappreciated host-participatory mechanism of biofilm development whereby bacteria exploit inflammation-induced endothelial remodeling to reinforce persistence. Moreover, pharmacological suppression and gene knockout of VIM substantially reduced E44 adhesion, invasion, and biofilm formation, consolidating VIM’s dual function as both the primary receptor and the upstream activator of NF-κB in IbeA-driven pathology (44, 45).

These mechanistic insights highlight the therapeutic potential of interrupting this upstream signaling transducer. Although traditional VIM inhibitors such as WA can effectively disable VIM, their off-target cytoskeletal toxicity limits its use in both clinical and pre-clinical studies (46, 47). In contrast, GA—a clinically approved immunomodulator with established neuroprotective effects—offers a viable strategy for safely disrupting this pathway (48, 49). Consistent with its known ability to suppress VIM expression, GA markedly reduced VIM expression in HBMECs, and attenuated IbeA-dependent adhesion, invasion, and biofilm formation. Importantly, in the neonatal rat model, treatment with GA improved survival, diminished bacterial burden, alleviated neuropathological injury, and preserved neurological function (48, 50). These results indicate that GA targets the VIM upstream signal transducer, effectively dismantling the inflammatory components of the IbeA-driven pathogenic cascade. Such dual-layer inhibitions offer a promising therapeutic strategy that overcomes the limitation of antibiotic treatment of infectious diseases.

Despite the progress made in this study, several important questions remain to be further investigated. First, genetic validation using Vim^-^/^-^ animal models will be essential to confirm the indispensable role of VIM during IbeA-dependent E44 infection *in vivo*, particularly in the initiation and progression of neonatal *E. coli* K1 meningitis. Second, given our findings that GA effectively blocks VIM in both *in vitro* and *in vivo* settings, the underlying mechanism needs to be defined—specifically, whether GA suppresses VIM expression or promotes its protein degradation. Understanding this mechanistic basis will be pivotal for rational optimization of VIM-targeted interventions. Moreover, considering the therapeutic benefits already demonstrated in this work, future studies should further evaluate the pharmacodynamic profile and translational potential of GA as a host-directed therapy against neonatal *E. coli* K1 meningitis. Third, interestingly, IbeA-mediated multiple virulence functions, including adhesion, invasion and biofilm formation, also contribute to the pathogenesis of avian infectious bacterial diseases caused by APEC strains. However, it remains to be addressed if the VIM–NF-κB signaling pathway plays a role in various diseases caused by APEC strains. Lastly, how IbeA and its regulon in GimA contribute to the pathogenesis of NMEC remains elusive. Our current fundamental research provides a solid foundation for future discoveries and innovations of GimA across various fields related to NMEC and APEC.

## 5 Conclusion

**Figure 7:**
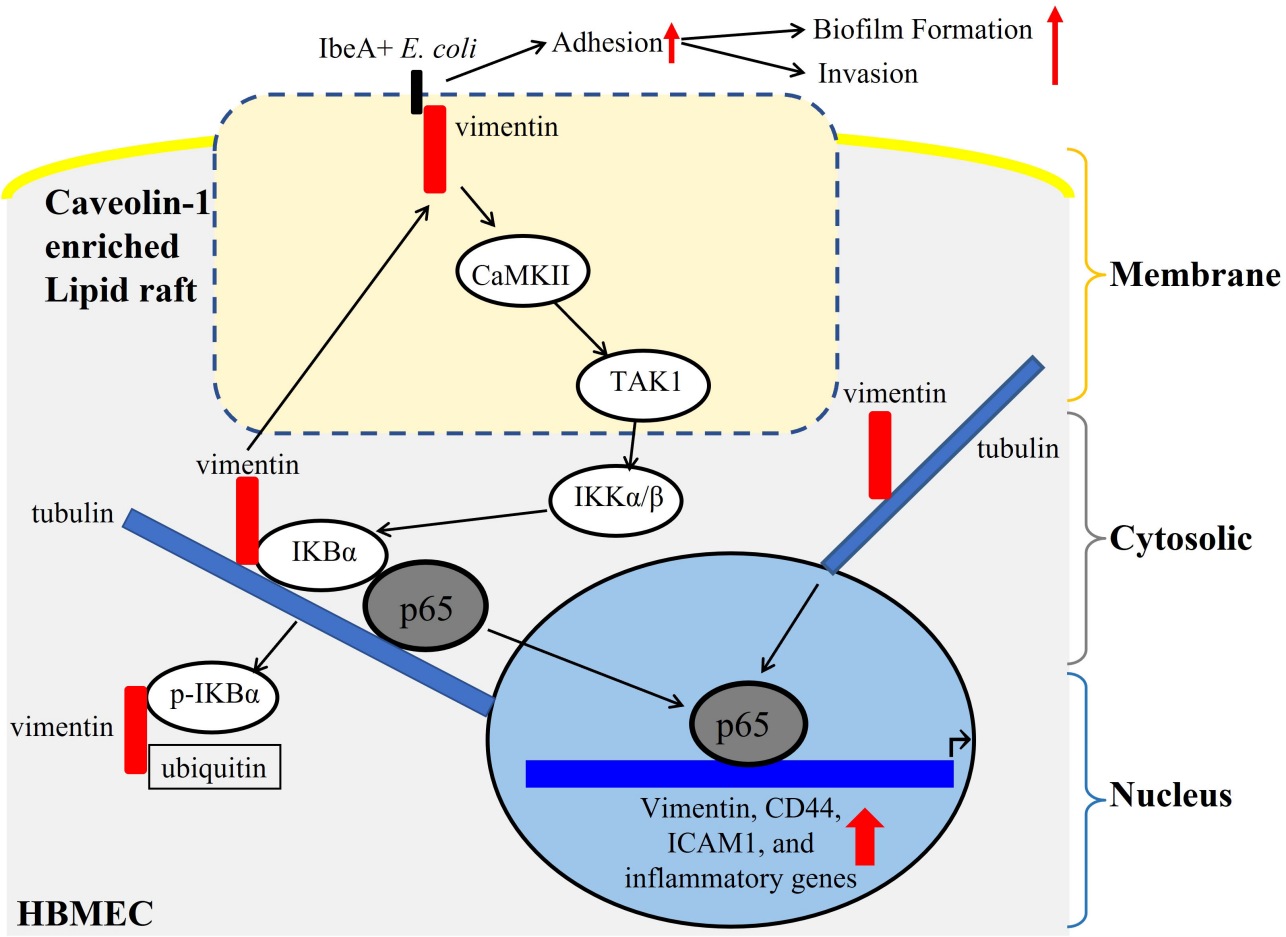
Schematic model of the VIM–NF-κB signaling pathway. **(A)** IbeA on *E. coli* K1 binds membrane vimentin within lipid raft microdomains, triggering VIM phosphorylation, citrullination, and cytoplasmic mobilization. **(B)** Activated VIM promotes NF-κB signaling by enhancing p65 phosphorylation and nuclear translocation. **(C)** NF-κB then induces transcription of CD44, ICAM1, and other proinflammatory mediators, driving HBMEC activation and injury. The host signaling pathway mediates *E. coli* K1 IbeA’s multiple virulence functions, including adhesion, invasion, and biofilm formation.

In summary, our study establishes IbeA, the most important pathogenic protein encoded by GimA, as a multifaceted virulence factor of *E. coli* K1 that coordinates HBMEC adhesion, invasion, and biofilm formation via orchestrated activation of the VIM–NF-κB pathway (**Figure 7**). By elucidating how a bacterial virulence factor interacts with the host defense and inflammatory pathways, we provide a unified mechanistic model for multiple virulence functions contributing to the pathogenesis of neonatal *E. coli* K1 meningitis. These insights support the development of new host-directed therapeutic strategies—exemplified by GA—that targets the upstream signal transducer VIM to mitigate disease and reduce long-term neurological sequelae.

## Data Availability

The original contributions presented in the study are included in the article/**Supplementary Material**. Further inquiries can be directed to the corresponding author.
