# Supplementary material for: Vimentin–NF-κB signaling contributing to IbeA-mediated adhesion, invasion, and biofilm formation during Escherichia coli K1 traversal of the blood–brain barrier
Source: Front Immunol. 2026 Apr 16;17:1793594. doi: 10.3389/fimmu.2026.1793594 (PMC13128420; doi:10.3389/fimmu.2026.1793594)
Supplement: Supplementary file 1 [file Table1.docx]

Supplementary Material 1

**Vimentin–NF-κB signaling contributing to IbeA-coordinated adhesion, invasion, and biofilm formation during *Escherichia coli* K1 traversal of the blood–brain barrier**

[1 Table. 1 modified neurological severity score 2](#_Toc223789579)

[2 Fig.S1 Bacterial adhesion and cell damage rate. 3](#_Toc223789580)

[3 Fig. S2 Representative HE staining of brain sections from neonatal rats infected with E44 or ZD1 4](#_Toc223789581)

[4 Fig. S3 Fluorescence images of IbeA and VIM observed by confocal microscope. 5](#_Toc223789582)

[5 Fig. S4 VIM deficiency impairs IbeA-mediated adhesion, invasion, and biofilm formation in HBMECs. 6](#_Toc223789583)

[6 Fig. S5 Effects of Cl-amidine and KN-93 on VIM modifications and E44-induced pathogenic phenotypes in HBMECs. 7](#_Toc223789584)

[7 Fig. S6 Effect of WA and GA on the expression of NF-κb in HBMEC infected by E44. 8](#_Toc223789585)

[8 Fig. S7 The cytotoxicity detection of GA and its impact on VIM expression in vivo. 9](#_Toc223789586)

# Table. 1 modified neurological severity score


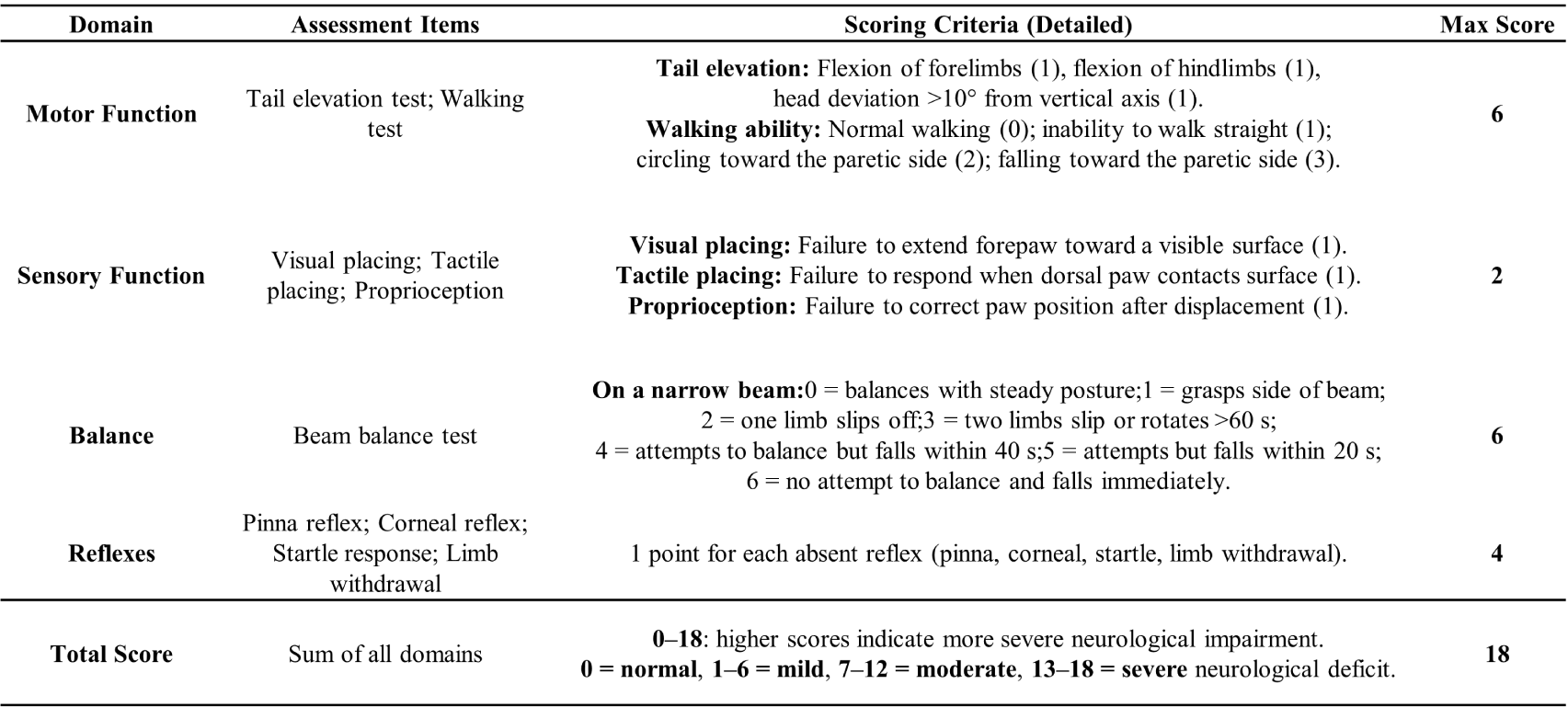


# Fig.S1 Bacterial adhesion and cell damage rate.


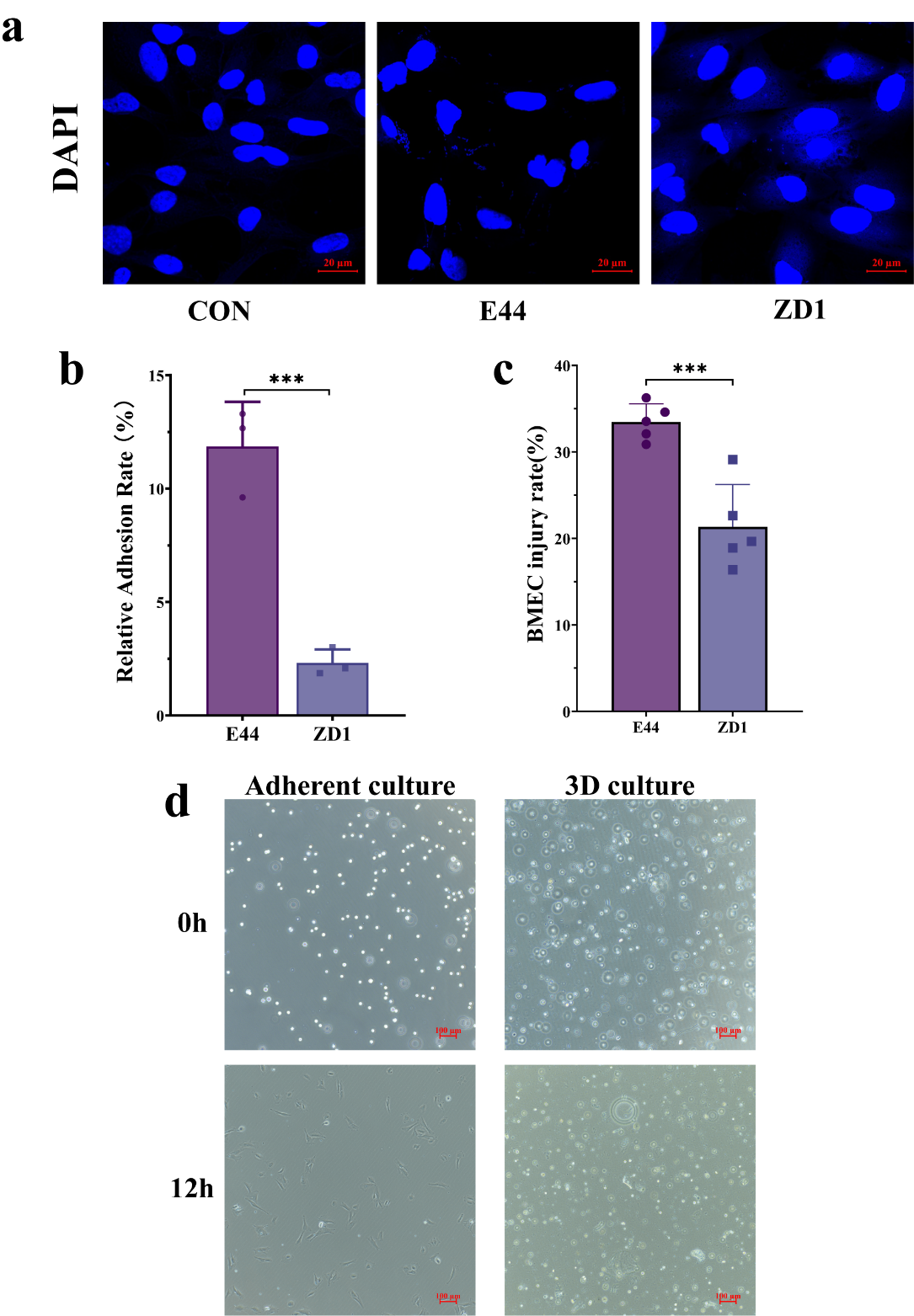


**a** After bacterial infection, DAPI was used to simultaneously stain the nuclei of bacteria and HBMEC, and the image under fluorescence microscope; **b** Quantified bacterial adhesion HBMEC ratio; **c** The damage rate of HBMEC after infection with two strains. **d** Representative brightfield images of monolayer and hydrogel-based HBMEC cultures. Data are from three independent experiments (mean ± SD). ***P < 0.001.

# Fig. S2 Representative HE staining of brain sections from neonatal rats infected with E44 or ZD1


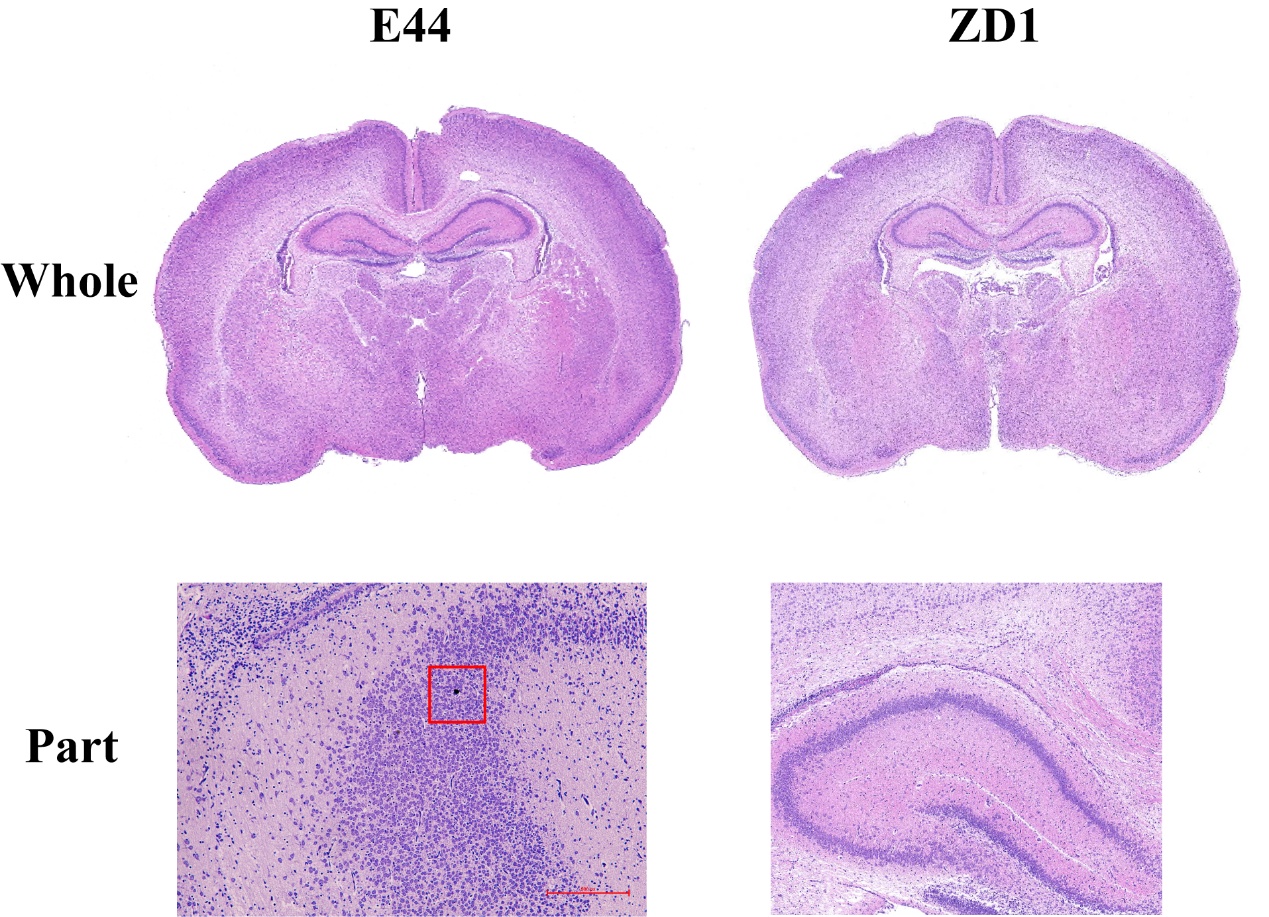


Brain tissues were collected from neonatal rats following infection with E44 or ZD1 and processed for HE staining under identical experimental conditions. Representative low-magnification images (upper panels) show overall brain architecture, while corresponding high-magnification images (lower panels) highlight local histopathological features.

# Fig. S3 Fluorescence images of IbeA and VIM observed by confocal microscope.


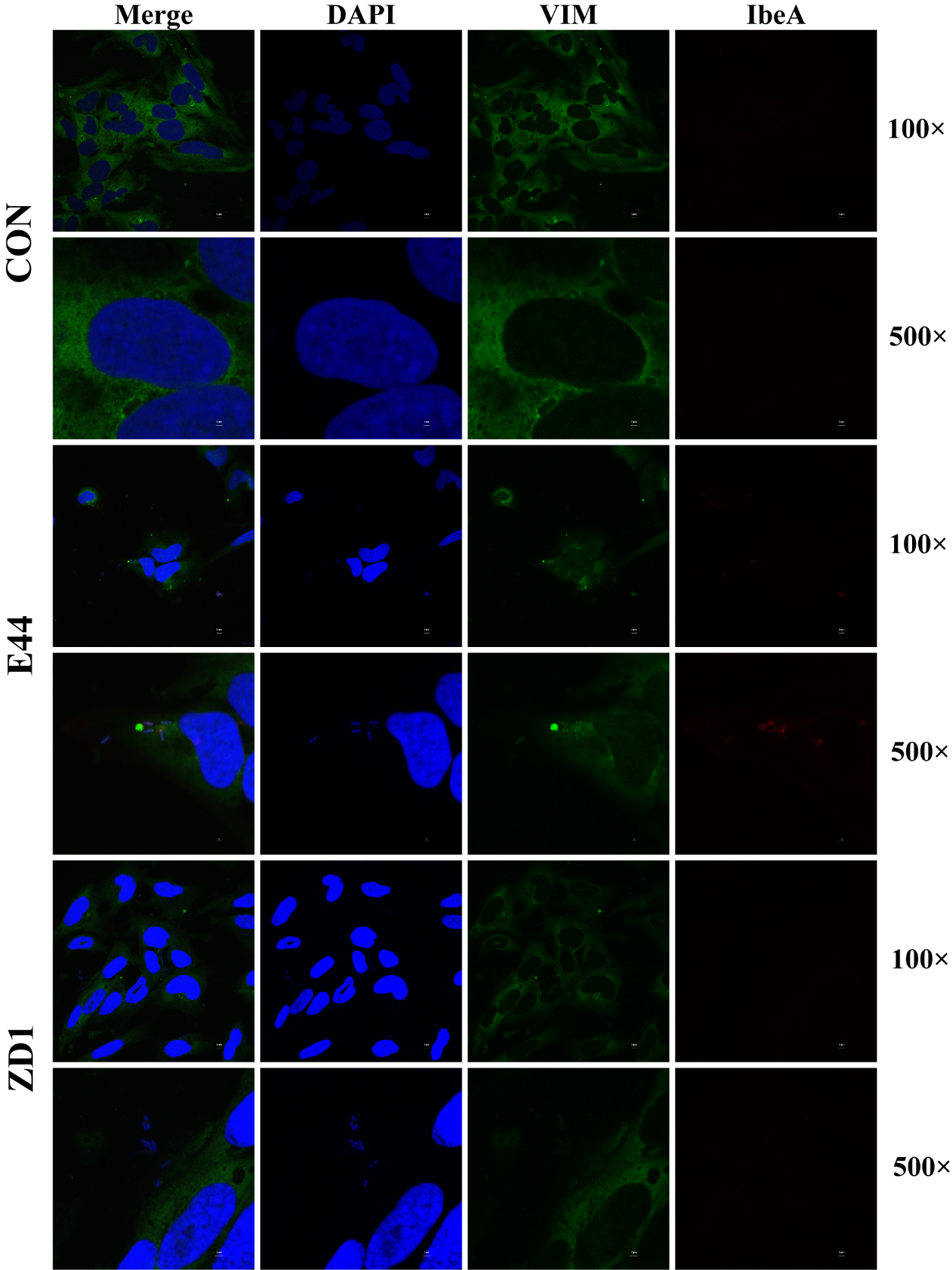


# **Fig. S4 VIM deficiency impairs IbeA-mediated adhesion, invasion, and biofilm formation in HBMECs.**


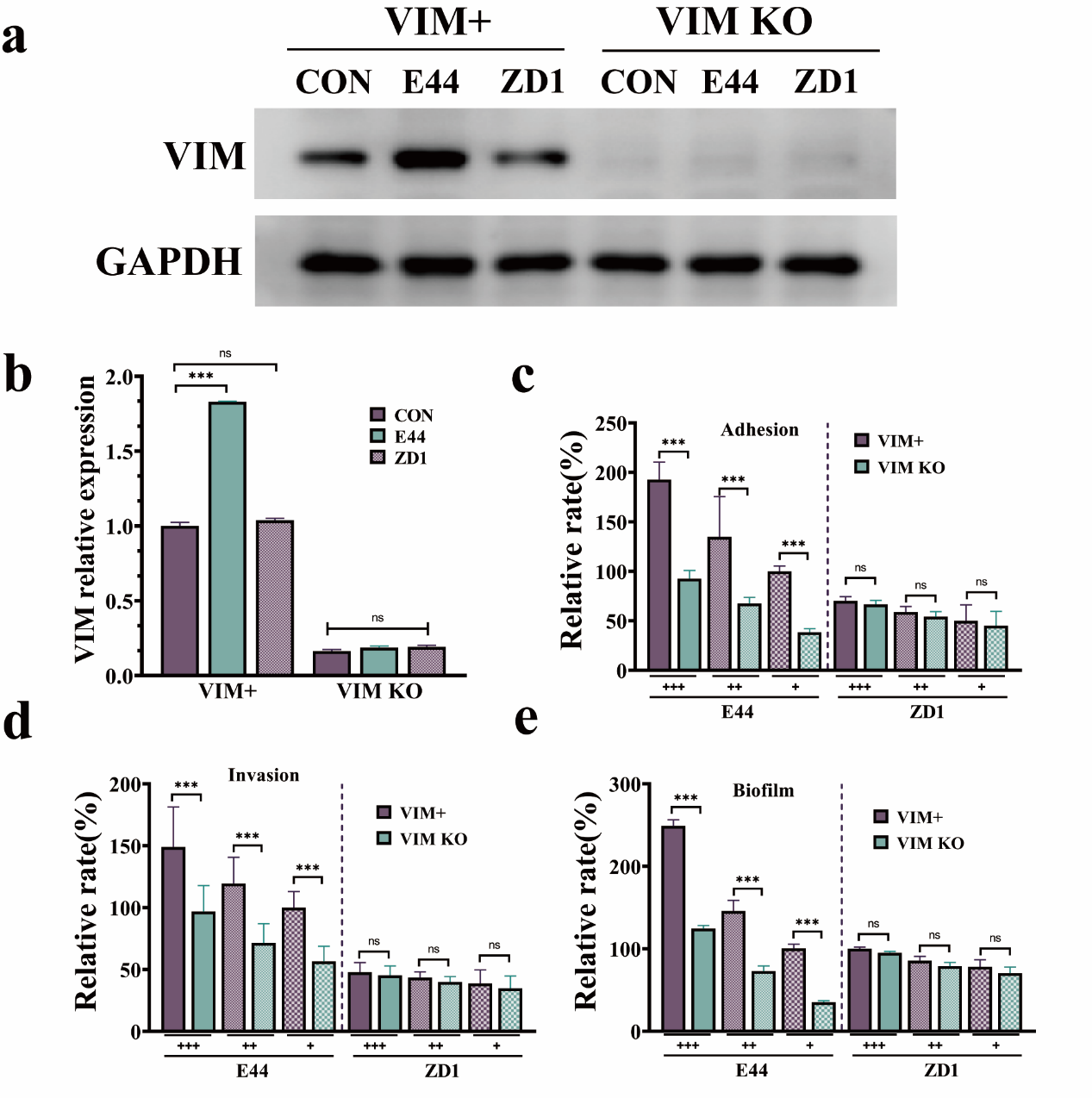


**a** Western blot analysis of VIM expression in wild-type (WT) and VIM knockout (VIM-KO) HBMECs following infection with *E. coli* strains E44 or ZD1; **b** Quantification of VIM protein levels shown in (a); **c** Bacterial adhesion assay of E44 and ZD1 in WT and VIM-KO HBMECs; **d** Bacterial invasion assay of E44 and ZD1 in WT and VIM-KO HBMECs; **e** Biofilm formation assay of E44 and ZD1 on WT and VIM-KO HBMECs. Data are presented as mean ± SD from three independent experiments.

# **Fig. S5 Effects of** **Cl-amidine and KN-93 on VIM modifications and E44-induced pathogenic phenotypes in HBMECs.**


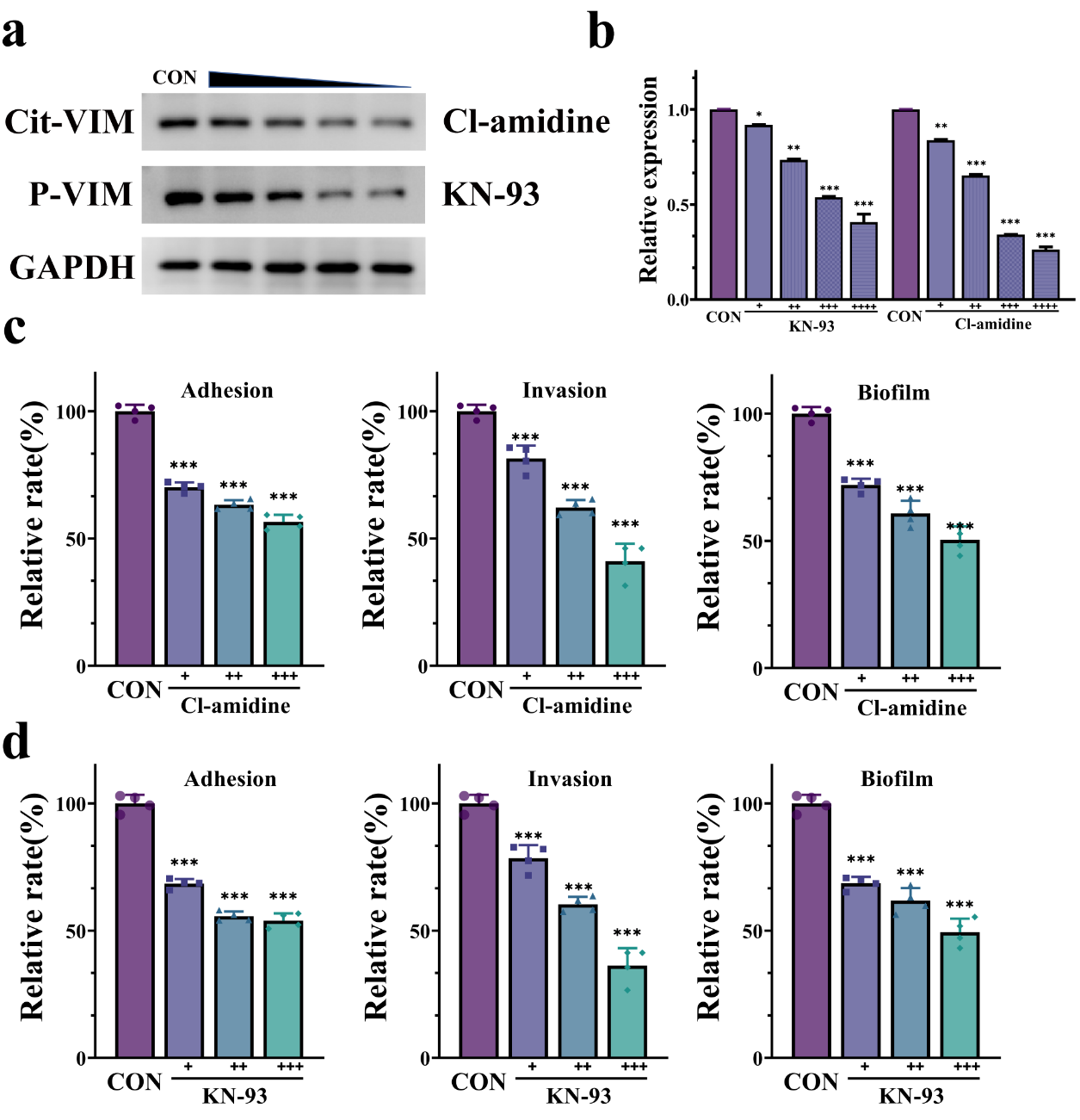


**a** Western blot analysis of VIM post-translational modifications in HBMECs treated with Cl-amidine or KN-93 followed by infection with *E. coli* E44; **b** Quantification of the VIM modification levels shown in (a); **c** Effects of Cl-amidine on E44-induced adhesion, invasion, and biofilm formation in HBMECs; **d** Effects of KN-93 on E44-induced adhesion, invasion, and biofilm formation in HBMECs. Data are presented as mean ± SD from three independent experiments. ***P < 0.001.

# **Fig. S6 Effect of WA and GA on the expression of NF-κb in HBMEC infected by E44.**


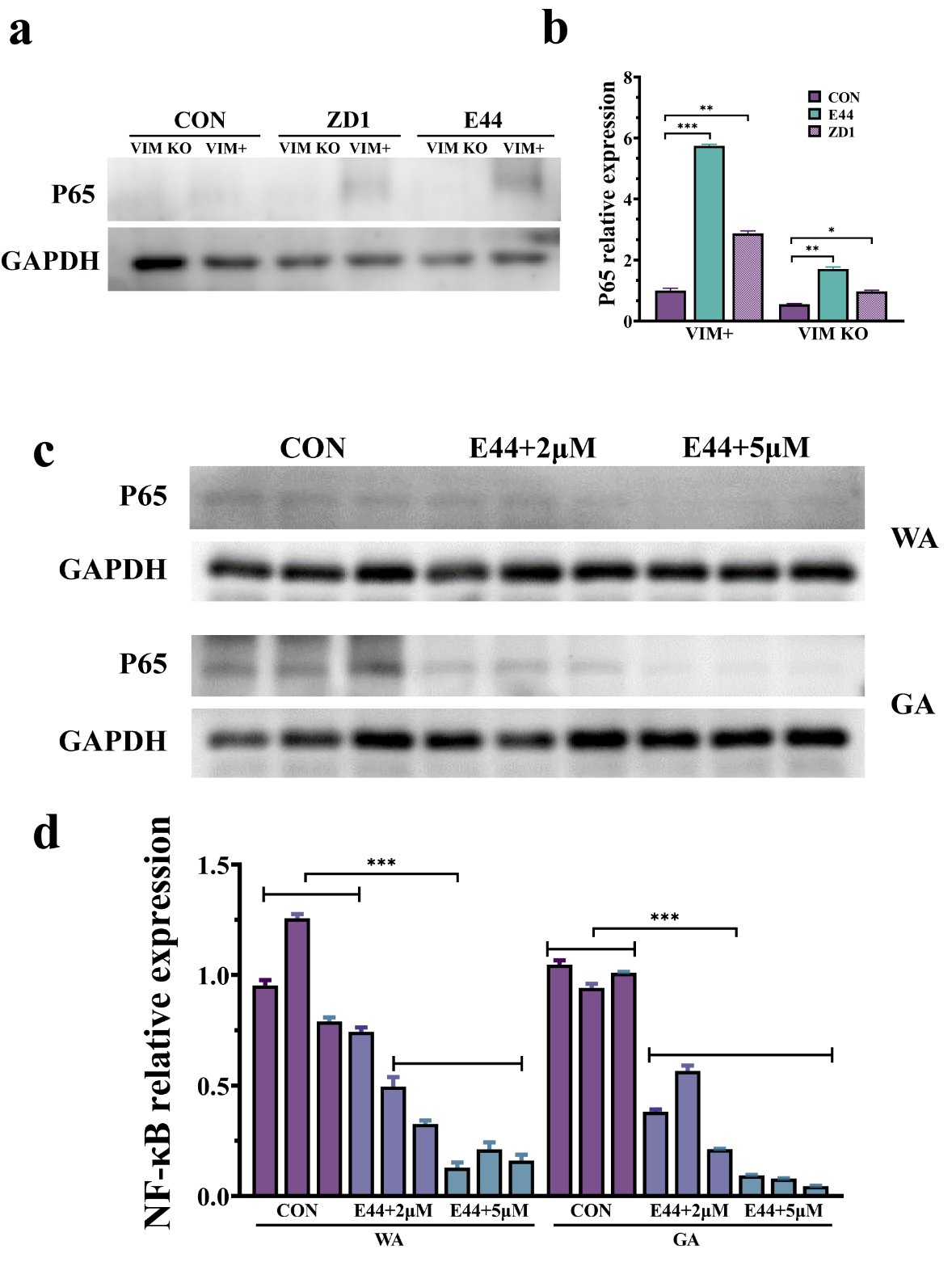


**a** Western blot analysis of NF-κB p65 in HBMECs treated with WA or GA followed by infection with *E. coli* E44; **b** Quantification of p65 protein levels shown in (a); **c** Western blot analysis of NF-κB p65 in wild-type (WT) and VIM-KO HBMECs following infection with *E. coli* E44; **d** Quantification of p65 protein levels shown in (c). Data are presented as mean ± SD from three independent experiments. ***P < 0.001.

# **Fig. S7 The cytotoxicity detection of GA and its impact on VIM expression in vivo.**


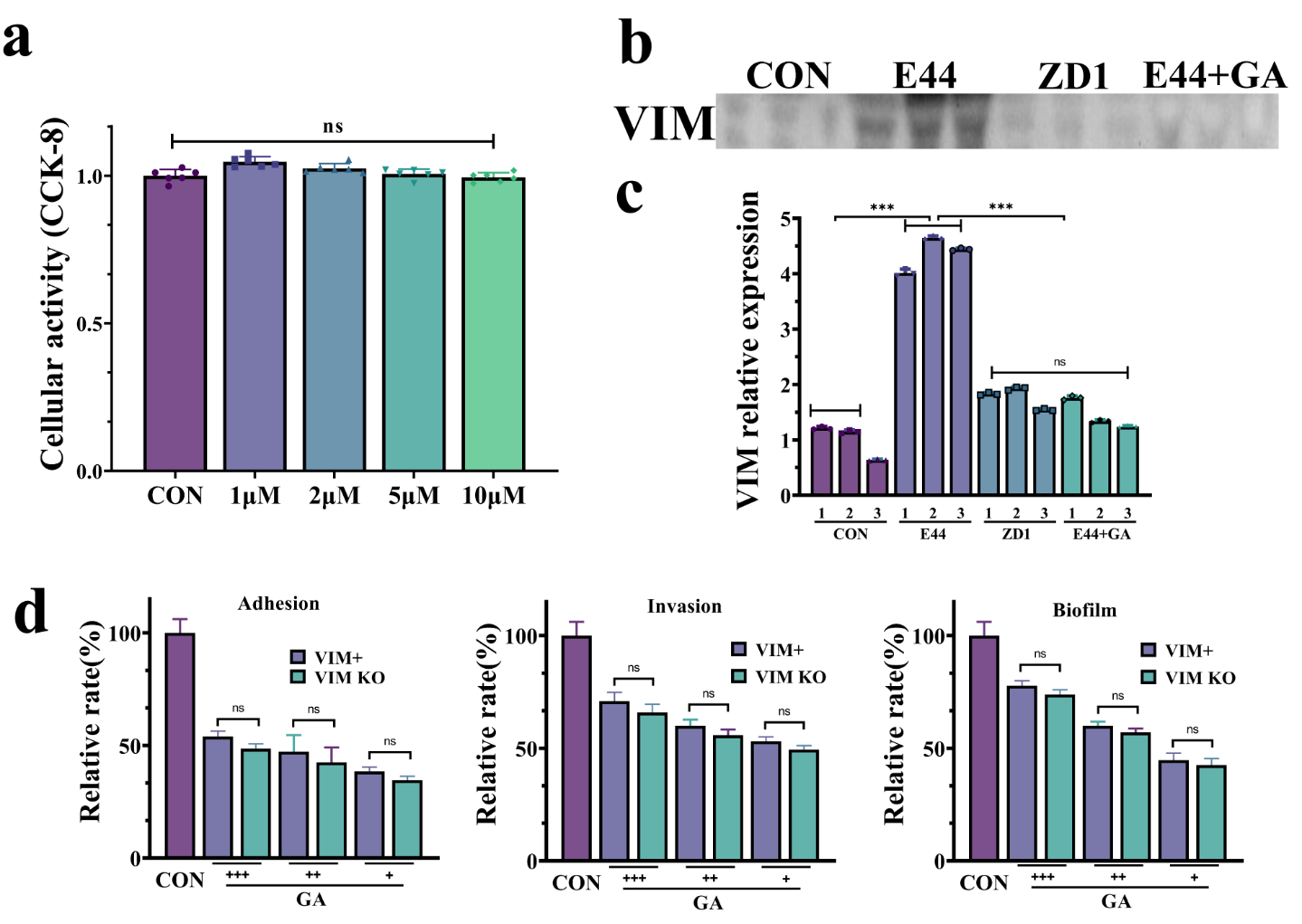


**a** Cell toxicity CCK8 detection of GA; **b-c** Changes in VIM protein in blood of rats with bacterial infection and GA treatment; **d** Adhesion, invasion, and biofilm formation assays of *E. coli* E44 in wild-type and VIM-KO HBMECs pretreated with GA. Data are from three independent experiments (mean ± SD). ns, not significant, ***P < 0.001.
