# Supplementary material for: Vimentin–NF-κB signaling contributing to IbeA-mediated adhesion, invasion, and biofilm formation during Escherichia coli K1 traversal of the blood–brain barrier
Source: Front Immunol. 2026 Apr 16;17:1793594. doi: 10.3389/fimmu.2026.1793594 (PMC13128420; doi:10.3389/fimmu.2026.1793594)

unedited blot/gel Fig. 3

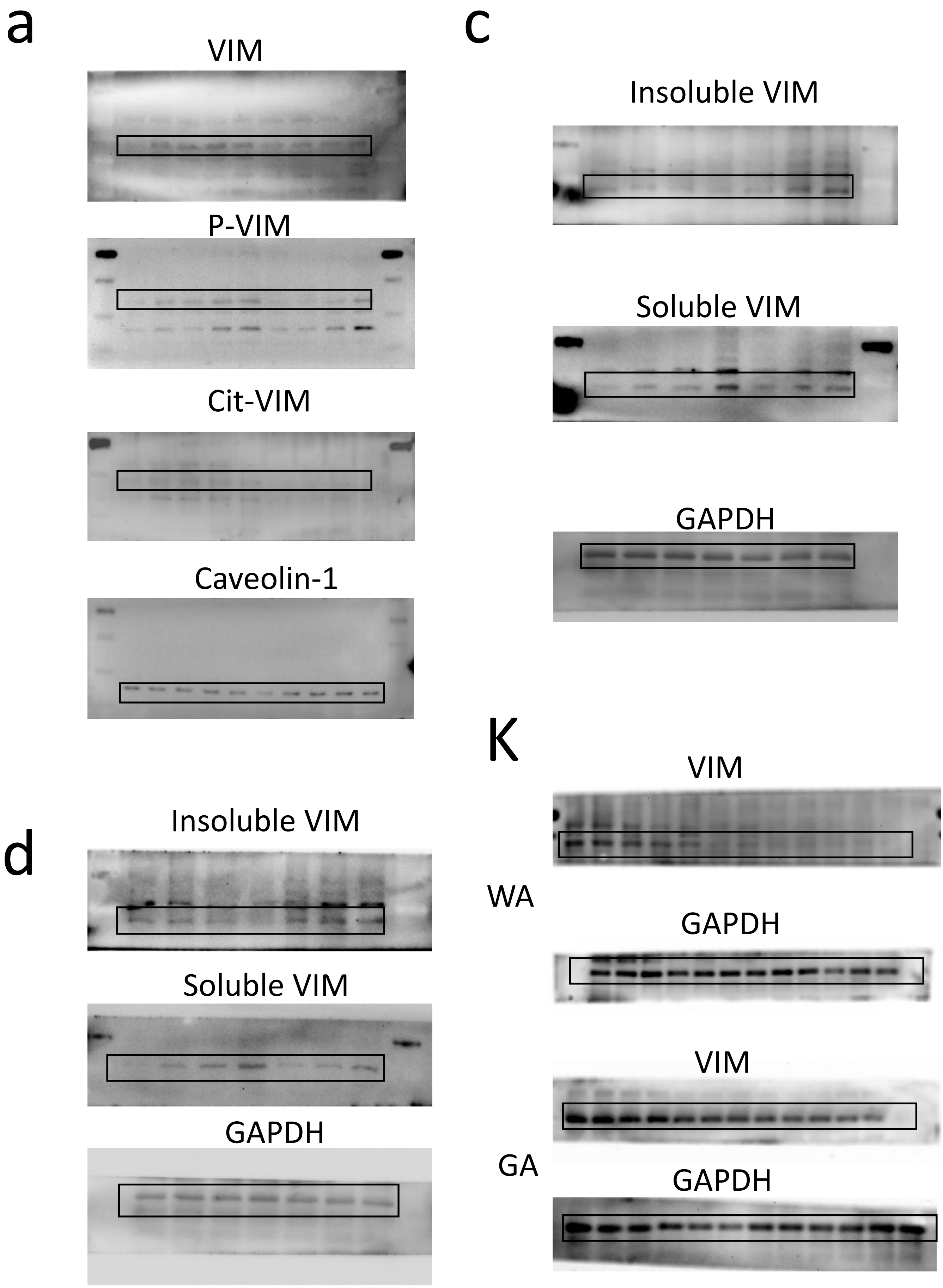

unedited blot/gel Fig. 4

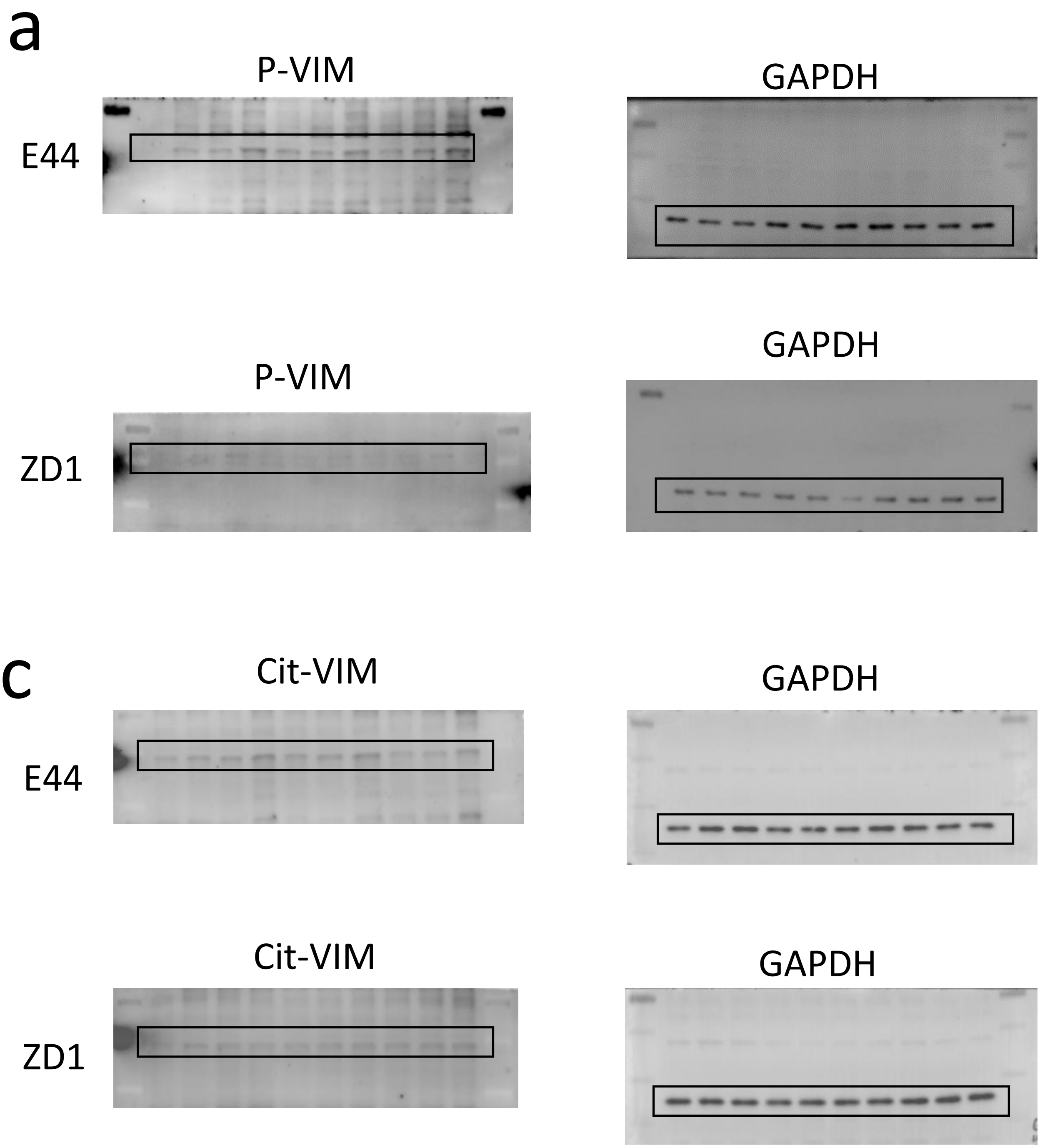

unedited blot/gel Fig. 5

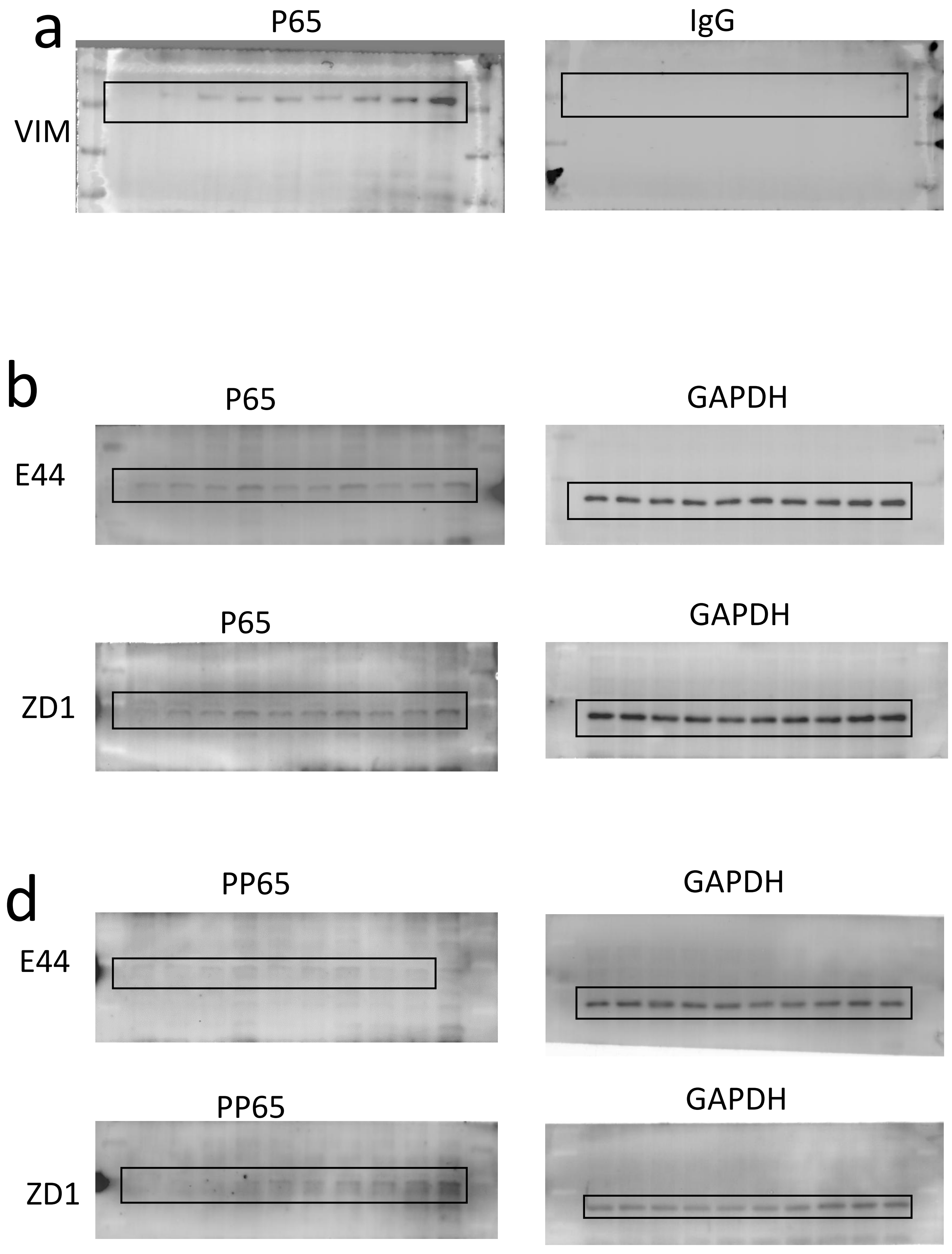

unedited blot/gel Supplemental Fig. S4

a

VIM

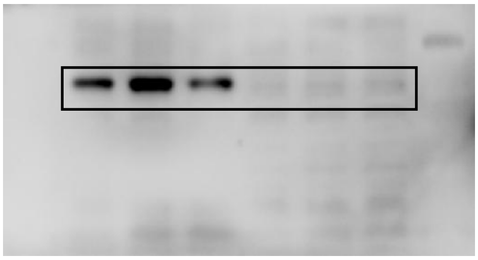

GAPDH

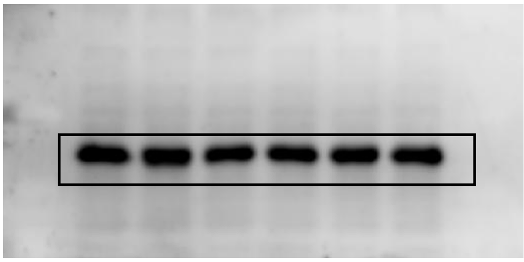

unedited blot/gel Supplemental Fig. S5

a

Cit-VIM

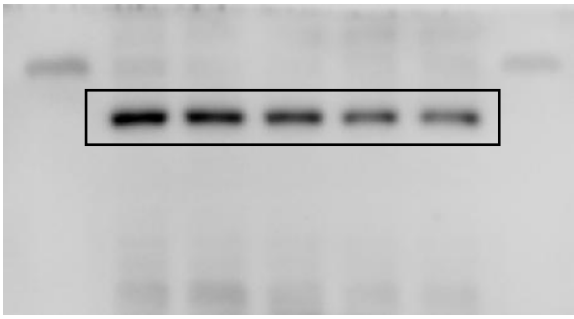

P-VIM

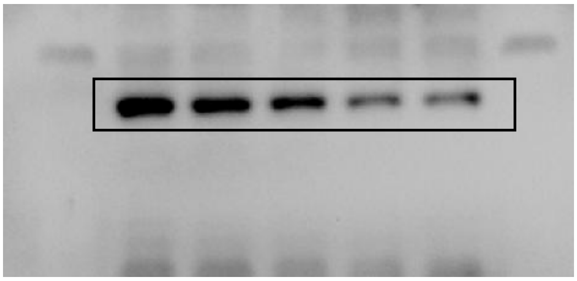

GAPDH

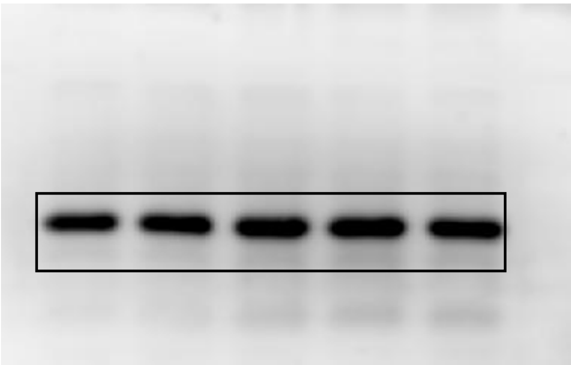

unedited blot/gel Supplemental Fig. S6

a

GAPDH

P65

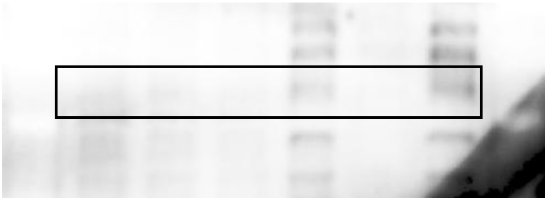

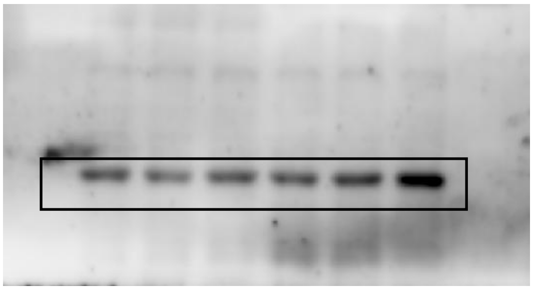

P65

c

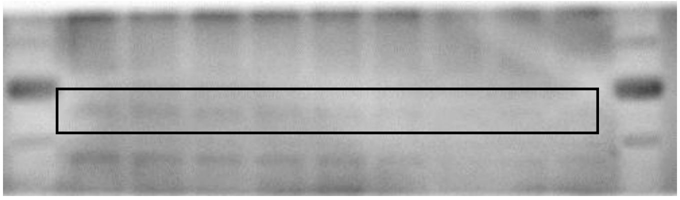

WA

GAPDH

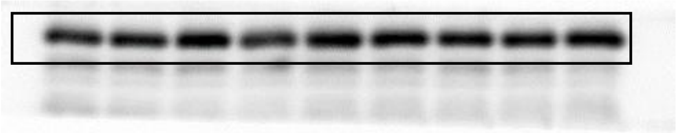

P65

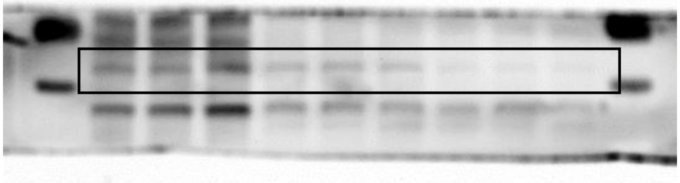

GA

GAPDH

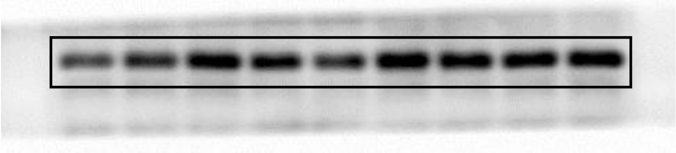

unedited blot/gel Supplemental Fig. S7

VIM

b

Blood

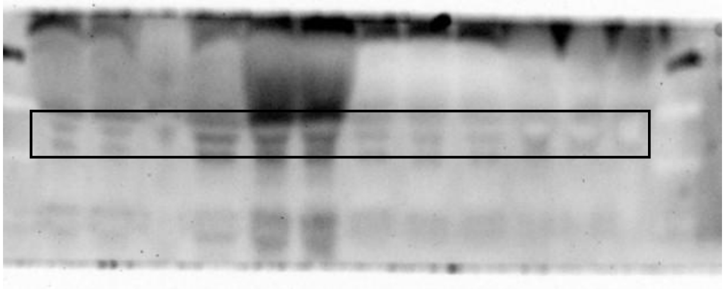

Supplement: Supplementary file 2 [file DataSheet1.pdf]
